# Regulatory approaches for genome edited agricultural plants in select countries and jurisdictions around the world

**DOI:** 10.1007/s11248-021-00257-8

**Published:** 2021-05-10

**Authors:** Jon Entine, Maria Sueli S. Felipe, Jan-Hendrik Groenewald, Drew L. Kershen, Martin Lema, Alan McHughen, Alexandre Lima Nepomuceno, Ryo Ohsawa, Reynante L. Ordonio, Wayne A. Parrott, Hector Quemada, Carl Ramage, Inez Slamet-Loedin, Stuart J. Smyth, Diane Wray-Cahen

**Affiliations:** 1 Genetic Literacy Project, Cincinnati, OH USA; 2 Genomic Sciences and Biotechnology Program, Catholic University of Brasília, Brasília, DF Brazil; 3 Biosafety South Africa, Somerset West, South Africa; 4 College of Law, University of Oklahoma, Norman, OK USA; 5 Departamento de Ciencia Y Tecnología and Maestría en Ciencia, Tecnología y Sociedad, Universidad Nacional de Quilmes, Bernal Buenos Aires, Argentina; 6 Botany and Plant Sciences, University of California, Riverside, CA USA; 7 General Head National Soybean Research Center - Embrapa Soybean, Embrapa Soja, Londrina, PR Brazil; 8 Faculty of Life and Environmental Sciences, University of Tsukuba, Tsukuba, Ibaraki Japan; 9 Crop Biotechnology Center, Philippine Rice Research Institute, Maligaya, Science City of Munoz, Philippines; 10 Department of Crop and Soil Sciences and Institute of Plant Breeding, Genetics and Genomics, University of Georgia, Athens, GA USA; 11 Department of Biological Sciences, Western Michigan University, Kalamazoo, MI USA; 12 Office of the Deputy Vice-Chancellor (Research and Industry Engagement), Rautaki Solutions Pty Ltd, La Trobe University, Melbourne, VIC Australia; 13 Fellow of The World Academy of Sciences, Cluster Lead-Trait and Genome Engineering, International Rice Research Institute, Manila, Philippines; 14 Department of Agricultural and Resource Economics, University of Saskatchewan, Saskatoon, SK Canada; 15 United States Department of Agriculture, Foreign Agricultural Service, Washington, DC USA

**Keywords:** Genome editing, Safety regulation, Regulatory approach, Crops and plants, International trade, Agricultural biotechnology

## Abstract

Genome editing in agriculture and food is leading to new, improved crops and other products. Depending on the regulatory approach taken in each country or region, commercialization of these crops and products may or may not require approval from the respective regulatory authorities. This paper describes the regulatory landscape governing genome edited agriculture and food products in a selection of countries and regions.

**Supplementary Information:**

The online version contains supplementary material available at 10.1007/s11248-021-00257-8.

## Significance statement

Genome editing techniques are rapidly being developed and applied to serve agricultural and food production objectives. In order to benefit fully, products developed using GEd must face reasonable, science-based safety regulations. This is particularly true of commodity crops, considering the proportion of such crops in international trade, and the prospect of their being subject to multiple, inconsistent and non-science based regulations as they traverse different jurisdictions. GEd crops developers need to be aware of the mosaic of regulations and regulatory schemes their products will have to pass prior to commercial release; this paper provides a glimpse of the varied approaches taken to regulating GEd crops in several jurisdictions around the world. For additional information, including ancillary data from several countries, the reader is directed to the Supplementary on line information accompanying this article. This article originally included a section on the EU, but revisions to that section were judged to be unacceptable by reviewers, who recommended rejection of the entire manuscript. In order to enable publication of the rest of the manuscript, the EU section was regrettably removed.

**Paul Christou**, *University of Lleida-Agrotecnio CERCA Center, Lleida, Spain and ICREA, Barcelona, Spain.*

## Introduction

This article provides an overview of proposed or adopted regulatory approaches in selected countries around the world for plants improved using genome editing (GEd) techniques. It describes the various directions taken by several countries, recognizing that other important trading countries, including, for example, China, have not released a specific regulatory approach tailored to GEd plants and their products. This article presents the most recent legal and regulatory developments in each jurisdiction described. The global landscape of regulatory developments for genome edited plants is rapidly changing and will continue to evolve as more countries release their regulatory policies. An overview of additional background information on the legal and regulatory frameworks for biotechnology and regulation of products of genetically engineered/modified plants in these jurisdictions is available in the supplementary information (SI) section. While not a comprehensive systematic collection, this review is meant to provide a broad overview of the various directions of regulatory approaches taken or under consideration in selected countries. It thereby adds to other recent reviews on the development of the regulatory landscape for GEd crops and updates or completes the information contained therein (Eriksson et al. 2019; Menz et al. 2020).

Genome editing is a generic term used to describe a host of methods for altering the genetic information in a cell, as described in other articles in this issue (see, for example, T-K Huang and H. Puchta. Novel CRISPR/Cas applications in plants—from prime editing to chromosome engineering in this Special Issue). Briefly, GEd encompasses several distinct types of alterations generating different products: site-directed deletions, allele replacement, site-directed insertions (or SDN-1/2/3 according to the terminology of Podevin et al. 2013) and base conversion (Marzec and Hensel, 2020). Some of these GEd processes involve insertions of DNA via the use of DNA templates (either cisgenic or transgenic) and others do not. These may each elicit a different regulatory approach, depending on the jurisdiction. Developers of new plant varieties improved using one or more of these ‘genome editing’ techniques face different research, legal, regulatory, and marketing requirements around the world. Adding further complications, different jurisdictions may apply different terminology.

In this paper, we use the term genetic engineering (GE) to refer to the use of recombinant DNA (rDNA) technologies to alter the DNA base sequence of an organism. GE technologies can be used to create a transgenic organism, which contains a genome consisting of DNA segments originating in different species. The modified organisms might also contain DNA segments originating in the same species but introduced through rDNA technologies, resulting in cisgenic organisms. The definition of a genetically modified organism (GMO) may vary between different jurisdictions; however, most countries have based their definition on the Cartagena Protocol on Biosafety (CPB) and its definition of a Living Modified Organism (LMO). The CPB defines a LMO as “any living organism that possesses a novel combination of genetic material obtained through the use of modern biotechnology.” The CPB also defines the terms 'living organism' and 'modern biotechnology'. Regulations, whether for conventional or biotech products, are intended to protect public health and safety, ensuring that products released into the market are as safe as possible for humans, animals, and the environment. Although all countries seek to promulgate regulatory approaches and processes to protect the common good of human, animal and environmental safety, the regulatory details in different jurisdictions can differ widely, and these differences and how they are implemented can have large impacts on the time required and cost of bringing new plant products of biotechnology to the global market place. Different laws and regulations for products of technologies using rDNA are in place around the world. The regulatory triggers for these products are generally based on the techniques used to create them, rather than the identification of any specific or novel potential hazards that such products may pose. While these laws and regulations differ among countries and regions, there is general agreement in each regulatory regime as to what products and processes are covered by these regulations for rDNA-derived products.

Divergent regulatory approaches may be a result of different economic, social and political prerequisites. Such divergence may not pose problems when applied to locally produced and consumed products (though time and cost of getting local products through the regulatory process could prove prohibitive). However, non-compatible, and unpredictable regulatory processes are problematic when applied to commodities entering into international trade, such as is the case for most agricultural biotech products currently on the market. Global trade in agricultural goods allows harvesting of economic benefits across regions. In order to facilitate such trade, globally harmonized or compatible regulations and policies can be an asset.

As GEd technologies emerged and started being used by developers and breeders of new plant varieties, regulatory authorities around the world began to examine their regulations and how these might apply to products improved with these new techniques. With the emergence of these new technologies, hope also emerged among breeders, researchers, and developers that with these new technologies new regulatory approaches would focus on the products developed and any risks they might pose, rather than the technologies used to create them. The previous global biotechnology regulatory landscape, which had general agreement as to what products required further regulation, has not been without its trade disputes,^1^ however the advent of GEd has introduced new challenges, especially with regards to regulatory distinctions and to traceability, potentially creating new types of regulatory and trade dilemmas. The sections below provide an overview of the different regulatory approaches being taken by several countries and regions in different parts of the world. It includes descriptions of the definitions or distinctions they are using to determine which plant products are included within the jurisdiction of their biotech or “GMO” regulations, with a special focus on recent developments.

## Jurisdictional considerations

### Canada

#### Introduction

Canadian research on genetically engineered (GE) crops was some of the earliest research to globally occur. Field trials with GE flax and canola began in 1986, resulting in Canada having 35 years of experience with regulating innovative plant breeding technologies (Smyth and McHughen 2008). Few other nations can claim such a lengthy period of innovative research and successful technology commercialization. The first two GE crops to receive regulatory approval were two herbicide tolerant (HT) varieties of canola, in March 1995. Over the subsequent 25 years, Canada has assessed the risks and commercially approved 123 crop varieties (CFIA 2020). These risk assessments are science-based and have proven the strength of the Canadian regulatory system, as no risk from the production of GE crops has been proven to differ from the risk of producing other conventional and non- genetically engineered crop varieties.

It is estimated that all of the canola planted in Canada is HT, as the last non-HT canola varieties to be reported in field trials occurred in 2012. Most of the canola produced in Canada is by GEHT varieties, 85–90%, with the balance being HT varieties developed by mutagenesis. Similar adoption levels are evident in corn production, as based on seed sales, in excess of 95% of seeded corn acres are done using the stacked traits of herbicide tolerance and insect resistance (Smyth 2014). Genetically engineered soybean adoption slightly lagged that of canola and corn, but in 2018, the average adoption rate for genetically engineered canola, corn and soybeans in Canada was estimated to 92.5% (ISAAA 2019).

#### Canada’s regulatory framework and impacts for GEd crops

Canada developed a product-based risk assessment framework known as plants with novel traits (PNTs), which regulates varieties, regardless of whether developed via mutagenesis, genetic engineering or GEd technologies. While novelty is not clearly defined, PNT regulations apply to a new plant variety with a trait(s) that expresses 25–30% higher or lower than the conventional variety. Further information on the regulations, requirements and risks that are assessed by Canada’s PNT regulatory framework can be found in the Supplemental Information. Health Canada has announced that new guidelines will be issued in April 2021, designed to add clarity to the application of PNT regulations to gene editing breeding technologies.

The risk assessment framework that exists for PNTs and governs the regulations of genetically engineered crop varieties, would apply to GEd crop varieties that are developed by public or private breeders and submitted for risk assessment and approval. The PNT regulatory framework would apply to a risk assessment regardless of whether it was a gene knockout, allele replacement or site-directed insertion. It would be the novelty of the phenotype/characteristics resulting from any of these genome edits that would be regulated, not what genome edits were undertaken to develop any new varieties. The main question regarding Canadian regulation of GEd plant varieties is whether, and how, the PNT regulatory framework would apply. Can herbicide tolerant canola be truly classified as ‘novel’, when no public or private plant breeder is commercializing non-HT canola varieties? Clarity regarding the definition of novel is essential as Canadian plant breeders increasingly adopt GEd technologies into their variety development programs.

The Canadian Food Inspection Agency (CFIA) has stated, dating back to the establishment of the PNT regulatory framework, that it is applied on a case-by-case basis and there is no reason to expect or believe that this approach would change if the variety was developed via GEd technology, as the CFIA has applied PNT status to varieties developed through the use of genetic engineering and mutagenesis. As an example, herbicide tolerant wheat was developed via mutagenesis breeding techniques and was regulated as a PNT. The only possible deviation from this regulatory approach, would be a blanket exemption, which could result should the CFIA rule that varieties developed through the application of GEd technologies that do not include any foreign DNA in the final variety, would be exempt from PNT regulation.

In an effort to encourage dialogue between regulators and the variety development sector regarding the potential to update PNT regulations pertaining to GEd, a workshop was held in 2017 to bring both groups together to discuss the advantages of Canada’s regulatory system and to identify opportunities for mitigating some of the concerns expressed by plant breeders (Canadian Seed Trade Association 2017). The workshop identified that GEd offers significant potential for the Canadian plant breeding sector, however reforms are required to capture the full value of these innovations. One key message from the workshop found that greater clarity regarding novelty is required, as plant breeders need to know what can be done within the existing gene pool without triggering novelty, especially for yield increases. This workshop resulted in Health Canada holding two years of industry roundtable consultations, with full public consultation to begin in January 2021 and a report on changes and/or improvements to be delivered in April 2021.

GEd technologies are being widely used in Canada, as a survey of nearly 100 public and private plant breeders about their use of clustered regularly interspaced short palindromic repeats (CRISPR) indicated. Gleim et al. (2020) found that 66% are using this technology, or plan to use it by 2021. Slightly more private breeders are open to using CRISPR compared to public breeders, 74% compare to 60%, respectively. When asked about the reason for choosing to use CRISPR to develop new crop varieties, the top response (90%) was easier pathway to regulatory approval. With such a high percentage of Canadian plant breeders using, or anticipating the use of a GEd technique like CRISPR, respondents were queried about whether the PNT regulatory framework required an update to better reflect the current state of plant breeding technologies, 77% of respondents indicated such an update was important (Smyth et al. 2020). Canada’s PNT regulatory framework is having a negative impact on plant breeding in Canada as one-third of respondents indicated they have terminated research projects upon self-determining the resulting variety would be a PNT. A review of the PNT regulatory framework is more topical for public breeder in Canada as, due to the additional costs of maintaining two separate breeding programs, very few public institutions develop varieties that will be deemed to be a PNT. Other bulk commodity producing countries, such as Argentina, Australia, Brazil and the United States, have indicated that if no foreign DNA is present in the variety, then the submitted variety would not be regulated as equivalent to a genetically engineered crop variety, thereby being regulated as equivalent to conventional crops (Smyth 2019). It is important to keep in mind that the presence or absence of foreign DNA (‘foreign’ is pejorative in this context), bears no relationship to the presence and/or absence of a novel hazard or any risk.

The challenge facing Canada’s PNT system is that the vast majority of private sector variety development companies operate in both Canada and the United States. The US has declared that if no plant pest properties have been changed, then GEd crop varieties will be regulated as equivalent to conventionally bred varieties (USDA 2018a). In a survey of plant breeders and regulatory experts, Lassoued et al. (2019) estimated that the time and cost to bring a GEd crop variety to market if regulated as equivalent to genetically engineered crop varieties would be 14 years and US$24.5 million, compared to 5 years and US$10.5 million if regulated as conventional varieties. A nine year commercialization lag poses a significant cost to the approval of GEd varieties and will serve as an investment deterrent, as Smyth et al. (2014) identified that regulatory delays of six years is sufficient to reduce the return on investment to the point that the private sector will no longer make investments in the development of new varieties.

The regulatory quandary created between the Canadian PNT approach and the American approach to GEd regulation in the absence of foreign DNA in the final variety is that firms developing varieties to be commercialized in both countries, may decide that the time and cost of obtaining variety approval in Canada does not warrant the investment, given the smaller size of the Canadian seed market. Over the past 25–30 years, Canada and the United States have invested significant efforts to harmonize regulations, to the point that now the same data required for risk assessment may be submitted to both Canadian and American regulators, regardless of where the field trials were conducted, provided agronomic and environmental conditions are similar between both destined production regions. To ensure that investments by multinational variety development firms remain attractive, Canada will need to harmonize its GEd regulations with those of the US.

With investments in the development of the major crop commodities becoming so globally competitive in nature, regulatory efficiency between Canada, the US, Brazil, Argentina and Australia takes on even greater importance. Canada’s use of novelty has placed it as unique among these nations in terms of the regulation of GM crop varieties and now that plant breeding is in the process of moving from gene insertion technologies to GEd technologies, Canada finds itself where the application of novelty is posing concerns for breeders. With three-quarters of Canadian public and private plant breeders indicating that a review and update of PNT regulations is required with the increasing use of GEd, it is evident that if this does not occur, Canada faces the likelihood of reduced investment in variety development.

Officials and regulators within the CFIA and Health Canada have been participating in roundtable dialogue events with plant breeders and the crop production industry, which is a very positive sign. If Canada is going to continue to attract international investments into future variety development projects, these discussions need to result in a revised regulatory framework that better clarifies novelty, providing assurance to investors that Canada is serious about removing, or at least reducing, regulatory barriers regarding increased use of GEd technologies. Canada should not abandon its excellent science-based regulatory system, but the regulatory system needs to be flexible enough to adapt to implement appropriate risk assessments for new technologies.

### Argentina

#### Introduction

The Argentine regulatory framework for agricultural biotechnology was initiated in 1991 when the National Commission on Agricultural Biotechnology (CONABIA) was created. The country then initiated the commercial release of GM crops in 1996, simultaneously with another five pioneer countries. Nowadays, Argentina is the third global grower of biotech crops (ISAAA 2018). Since 2014, the Food and Agriculture Organization of the United Nations (FAO) has recognized CONABIA as its Center of Reference for Biosafety of Genetically Modified Organisms (FAO 2014).

Therefore, the Argentine regulatory system is one of the more seasoned ones regarding safety assessment practice, experience in the commercial adoption of agricultural biotechnology, and leadership in developing regulatory criteria.

Regulatory assessment of products of new plant breeding techniques including genome editing. In 2012, Argentine regulators noted the increasing relevance of innovative breeding techniques in the scientific literature, as well as an influential report of the European Joint Research Center on them (Lusser et al. 2011). Therefore, a policymaking process began to have regulatory criteria in place by the time products improved with these techniques would reach the regulators’ desks.

The regulators began by discussing if products from NBTs were within the scope of the GMO definition (which Argentine regulation took from the Cartagena Protocol). For this purpose, several examples of the scientific literature were used as case studies. They concluded that many products were not GMOs on a case-by-case assessment. They then discussed the gap between the agricultural biotechnology regulation and the regulation of conventional new varieties, since the registering of GEd products would involve both. Finally, regulatory processes were designed to balance the developers´ need for early certainty on their products’ regulatory stance, vis-a-vis the requirement of end-product data for their assessment.

By 2015, regulators of North American and European countries had made several decisions on the regulatory status of specific products. However, no model criterion of universal applicability was still available. That year, Argentina enacted the first regulation worldwide to establish a decision-making process for determining if products obtained with the aid of these technologies should be regulated as GMOs or not, on a case-by-case basis. The overall characteristics of this regulatory approach are described in Whelan and Lema (2015).

#### Practical experience

So far, a couple of dozen products have been presented for clarification of their regulatory status. With a few exceptions, most of them were considered non-GMOs. A majority are products from GEd, although other innovative breeding techniques are also present. This experience confirmed that the regulation works in practice and adapts to different technologies, traits, and organisms (Lema 2019).

The majority of developers are from the public research sector and national biotechnology enterprises that have never before submitted an application to the regulatory system. However, they were able to navigate the consultation process successfully. The developer and product profiles observed in Argentina have interesting implications regarding innovation economics analyzed elsewhere (Whelan et al. 2020).

The experience gained in assessing GEd products also provided the opportunity to help in clarifying issues under debate, such as the regulatory criteria that should be applied regarding off-target modifications and spurious DNA insertions (Lema 2020).

#### Communication with the public and other governments

The Argentine authorities organized workshops for the general public and communicators in which GEd developments and their regulation were debated. Also, socioeconomic studies on the impact of GEd have been outlined (Whelan and Lema 2017). The public´s responses display confidence towards the national regulatory agencies; they also reflect a positive interest in products under development, seemingly because of their local origin and traits addressing consumer and environmental benefits.

The Argentine Government has also engaged in discussing how to reach a harmonized approach at the international level; for instance, by fostering exchanges about the issue in the WTO Committee on Sanitary and Phytosanitary Measures Committee (SPS 2018), G-20 (MACS Argentina 2018), OECD, and bilateral fora (Argentina 2019a, 2019b).

#### Other regulations in the region

After the Argentine regulation was enacted, other Governments in the region took similar initiatives. The first to follow was Chile in 2016, then Brazil and Colombia in 2018 (Whelan and Lema 2019), and finally Paraguay, Ecuador, Honduras, and Guatemala in 2019. Also in 2019 Ecuador clarified in its internal regulations that only those organisms harboring recombinant or foreign DNA would be considered GMOs (thus implicitly excluding SDN1 and cisgenesis). The reasons why these were the first countries to take a stance on GEd seems to be purely domestic; they relate to the maturity of their regulatory systems and an interest in providing a predictable and enabling environment to ongoing local projects.

The regulations issued by Chile and Colombia strongly resemble the Argentine text. The Brazilian regulations, which are described in the next section, have a different text. However, an analysis of its application to concrete examples shows that it leads to the same conclusions as the Argentinean regulation (AgroLatam 2018).

Paraguay, Ecuador, Honduras, and Guatemala have issued regulations that enable excluding GEd products from GMO regulation, although written with a lesser amount of explicit details. However, their spirit seems to point in the same direction as the other countries that preceded them. More countries in the region are currently considering enacting their GEd regulations inspired by these precedents (Gatica-Arias 2020).

### Brazil

#### Introduction

The principles used to elaborate the Brazilian Biosafety Law (No. 11.105 of 24 March 2005) were to encourage scientific advances in the areas of biosafety and biotechnology, protection of life, human health, animal and plant health and compliance with the precautionary principle for protection of the environment, according to Cartagena Protocol on Biosafety (Secretariat of the Convention on Biological Diversity 2000). Its purpose and scope were to provide safety standards and inspection mechanisms for the construction, cultivation, production, handling, transportation, transfer, import, export, storage, research, environmental release and commercialization of GMOs and their by-products. CTNBio through its Normative Resolutions is responsible for establishing the biosafety guidelines for subjects of its competencies. Among its prerogatives and in consequence of the development of science and technology in the world, the law mandates the CTNBio to evaluate how new technologies can impact the environment, and human and animal health in the country and then, if necessary, authorize the commission to propose regulations for these new technologies. CTNBio consists of 27 Brazilian citizens, appointed by the Minister of Science and Technology (S&T), who have recognized technical competence, outstanding scientific performance and knowledge, an academic degree of doctor, and outstanding professional activity in the fields of: Biosafety, Biotechnology, Biology, Human Health, Animal Health, and Environment.

As with many other plant breeding techniques, the use of GMOs in agriculture has become important for the production of food and food products. However, unlike other technologies, the regulatory frameworks that support these outputs are based on an extensive list of requirements for a risk assessment that differ from country to country. Nevertheless, in many cases the requirements are not proportional to the risks, resulting in costly and time-consuming regulatory approval processes. As a consequence, only a few large multinational corporations have adequate resources to have new GM crops approved, while publicly funded research laboratories and small and medium-sized institutions/companies usually are unable to develop a GM product that can reach the market. In recent years, however, after more than two decades of experience, legislators have had the opportunity to learn from the experience gained with GMOs and how to create effective regulatory milestones for some emerging technologies such as those of GEd. The Brazilian 2005 Biosafety Law, although 15 years old, gives to the National Biosafety Technical Commission (CTNBio) the mandate to monitor the development and technical-scientific progress attained by biosafety, biotechnology, bioethics and related areas, and propose new legislation maintaining biosafety standards, but at the same time allowing technological development in Brazil. The Brazilian normative for New Breeding Technologies, specifically, GEd, is under this scope.

#### New breeding technologies under the Brazilian biosafety law

In Brazil the CTNBio has the mandate to evaluate how new technologies might impact biosafety for the environment and human/animal health and then, if necessary, to propose legislation regarding these new technologies. When the law was created, most of the New Breeding Techniques (NBT; know in Brazil as TIMP, from the Portuguese terminology: *Técnicas Inovadoras de Melhoramento de Precisão*), were at their infancy, so they were not really considered at that time. Thus, in 2014 the CTNBio created a working group of experts that studied these new breeding techniques, such as GEd, for three years. The aim was to propose a more updated normative under the scope of the Brazilian biosafety law. The CTNBio’s Normative Resolution n^o^16 (RN16) was then published on 15 January 2018 (RESOLUÇÃO NORMATIVA Nº 16, 2018). It was approved by unanimous vote by the CTNBio’s 27 members. The normative, which is also based on other countries’ experiences, evaluates in a case-by-case consultation system if a product generated by the NBTs will be considered a conventional or a transgenic organism. Under the RN16 consultation procedure, developers provide information on the product, including the methods used to generate it. The absence of recombinant DNA/RNA in the progeny, the presence of genetic elements that could be obtained by conventional breeding; the presence of induced mutations that could also be obtained by older techniques, such as radiation or chemical mutagenesis, or even the presence of induced mutations that could occur naturally, are analyzed on a case-by-case basis, and could be considered conventional organisms/products in many situations.

In practical terms, products obtained either by site-directed random mutation involving the joining of non-homologous ends (SDN1 mutation), or site-directed homologous repair involving one or few nucleotides (SDN2 mutation) meets the conditions established in Normative Resolution No. 16 to be designated as non-GMO in a case-by-case analysis. In contrast, site-directed transgene insertions (SDN3 mutation) are designated GM according to the provisions of the resolution. If the product is designated as GMO the developer will have to go through all the biosafety requirements and will be approved only after the CTNBio risk assessment. If the product is designated non-GMO, it can be registered through the existing procedures for conventional products. The CTNBio Normative Resolution n^o^ 16 is applicable to all types of organisms, including plants, animals and microorganisms, and can be considered at any stage of development.

#### Impact of policy on innovation

CTNBio's normative RN16 has enabled emergence of new companies (sstartups) and the strengthening of medium and large national companies in the development of biotechnological products and solutions for agribusiness, industry and animal/human health (Hua et al. 2019; Li et al. 2020; Zhao et al. 2019). Many Brazilian young scientists now are entering the job market with innovative companies offering solutions especially for Brazilian agribusiness. As of September 2020, there were 23 consultations with CTNBio. According to the provisions RN16, the crop products analyzed were all considered by CTNBio to possess the characteristics established in the Normative and were not considered to fall under the scope of the Law 11.105/2005 that regulates genetically modified organisms in Brazil. Also, small companies are now stimulated and aiming to develop products using new technologies in crop breeding considering this new regulation for biotechnology.

### United States

#### Introduction

The United States took a different approach for biotechnology regulation than most other countries by not creating any new laws specific for biotechnology. In 1986, the U.S. Office of Science and Technology Policy (OSTP), an office of the White House, published the Coordinated Framework for Regulation of Biotechnology (OSTP 1986), a policy for research and products of biotechnology that drew on existing laws to establish a forward-looking regulatory framework. [See Supplemental Information for additional details on the U.S. Coordinated Framework].

The Coordinated Framework states that Federal regulatory oversight would be risk-based and focus on the characteristics of the biotechnology product, rather than on the genetic modification technique used to create it. OSTP issued its most recent update of the Coordinated Framework in 2017 (OSTP 2017). Recent updates to the Coordinated Framework and U.S. regulatory processes to accommodate products produced via new biotechnologies such as GEd, were prompted by two separate presidential actions.

The first action was the “*Memorandum on Modernizing the Regulatory System for Biotechnology Products*” issued by the Executive Office of the President of the United States in July 2015. It directed the primary agencies responsible for regulating the products of agricultural biotechnology (USDA, EPA, FDA) to update regulatory roles and responsibilities under the Coordinated Framework for the Regulation of Biotechnology and also to develop a long-term strategy to ensure that the federal biotechnology regulatory system is prepared for future products of biotechnology. The “*National Strategy for Modernizing the Regulatory System for Biotechnology Products*” was released in 2016 (OSTP 2016) and the “*Update to the Coordinated Framework for the Regulation of Biotechnology*” was finalized in 2017 (OSTP 2017).

The second action was the “*Executive Order (EO) on Modernizing the Regulatory Framework for Agricultural Biotechnology Products*” issued by the President in June 2019. This EO called for U.S. regulatory agencies to review their authorities, regulations, and guidance and to take steps to update them. The biotechnology EO specifically mentions encouraging agricultural innovation and regulatory streamlining, directing agencies to use existing statutory authority, as appropriate, to exempt low-risk products of agricultural biotechnology from undue regulation (EO 13874 2019). The USDA has since updated its procedures and issued a Final Rule for its biotechnology regulations (*SECURE rule on Movement of Certain Genetically Engineered Organisms*) and the EPA, as of this writing, has proposed a rule for “*Exemptions of Certain Plant-Incorporated Protectants (PIPs) Derived from Newer Technologies*”.

#### United States Department of Agriculture (USDA)

USDA’s Animal and Plant Health Inspection Service (APHIS) is responsible for protecting U.S. agriculture, environment and economy from pests and diseases. APHIS has regulatory authority over certain plants and plant products of biotechnology under the Plant Protection Act (PPA), which obligates USDA to protect plant health. Plant pest risk is the potential to cause direct or indirect injury to, damage to, or disease in plants or plant products resulting from introducing or disseminating a plant pest, or the potential for exacerbating the impact of a plant pest.

In 1987, APHIS issued regulations for products of biotechnology under PPA authority over the importation, inter-state movement, or release into the environment of any “regulated article”. At that time, breeders of genetically engineered plants most commonly used *Agrobacterium tumefaciens*-mediated gene transfer and vectors containing rDNA sequences of bacterial or viral origin (both plant pests) and based on definitions in the regulations these genetically engineered plants were “regulated articles.”

By 2010, developers of plants that had been created using breeding technologies that did not use a plant pest to deliver DNA (e.g., direct DNA transfer) or did not retain DNA from a plant pest in the final plant product began sending APHIS letters of inquiry as to whether a particular plant was regulated. In response to these queries, APHIS created an “Am I Regulated?” (“AIR”) process. By October 2020, APHIS responded to over 168 letters of inquiry stating in each case that the particular plants created using different biotechnologies were “not regulated.” Over 90 of these responses have been for plants created via GEd. For example, Calyxt, Inc. received a “not regulated” letter from APHIS for several crops developed using the TALEN GEd technology (Calyxt 2020).

After years of regulatory experience with plant products of biotechnology, advances in biotechnology, including the emergence of GEd (e.g., site-directed nucleases—SDN) technologies in the 2010s, APHIS initiated efforts to update and revise its biotechnology regulations under the PPA. In 2020, APHIS issued revised regulations for organisms produced using biotechnology (USDA-AsPHIS , 2020). To better understand the APHIS Sustainable, Ecological, Consistent, Uniform, Responsible, Efficient (SECURE) rule, we will contrast it with the earlier APHIS regulations mentioned above and further described in the Supplemental Information (SI).

The SECURE rule applies to any genetically engineered organism that is or may pose a plant pest risk. The rule expands the definition of genetic engineering beyond the use of rDNA to include the use of nucleic acids (not just DNA) that have been synthesized or amplified to modify or create a genome. The APHIS SECURE rule applies to organisms modified using GEd as well as rDNA technologies.

APHIS introduced several exemptions for certain modified organisms that could be created through conventional breeding, as plants are not a priori a plant pest risk just because their development involved molecular techniques. The APHIS exemptions are for single genetic modifications if there is: (1) “a change resulting from cellular repair of a targeted DNA break in the absence of an externally provided repair template”; or (2) “a targeted single base pair substitution”; or (3) “a gene known to occur in the plant’s gene pool, or makes changes in a targeted sequence to correspond to a known allele of such a gene or to a known structural variation present in the gene pool.” If the allele inserted via editing is not known to occur in the plant’s gene pool, a regulatory status review will be necessary to ensure the resulting plant does not pose a plant pest risk. The single targeted genetic criterion for categories 1 and 2 applies only to both alleles of a locus in a pair of chromosomes; it does not extend to homoeologous alleles in a polyploid plant. Based on years of experience with certain genetically engineered organisms, APHIS will continue to exempt genetically engineered *Arabidopsis thaliana* from permit requirements and added exemptions for genetically engineered disarmed *Agrobacterium* species and genetically engineered *Drosophila melanogaster*.

Regardless of whether a DNA sequence is altered via traditional transformation or via GEd, and in a significant departure from past practice, APHIS has ended its event-by-event approach and changed its regulation to focus on the “mechanism of action (MOA)” (i.e., “the biochemical process(es) through which genetic material determines a trait”). Under the SECURE rule, once APHIS has determined that a given genetically engineered plant developed using a gene that works via a particular MOA is not subject to regulation as a plant pest or posing a plant pest risk, then new genetically engineered events of that plant using any gene with the same MOA are not subject to further requirements and permits under the biotechnology regulation. As an example of the focus on the MOA, consider the following: APHIS has approved the MOA of a glyphosate-resistant crop through the use of a glyphosate-insensitive EPSPS gene. (EPSP synthase, 5-enolpyruvylshikimate-3-phosphate synthase, is a crucial enzyme produced by plants and microorganisms and target of the popular herbicide glyphosate). That particular glyphosate-insensitive EPSPS gene would be exempt from further regulation in additional events of that crop. However, if a developer created a glyphosate-resistant version of that same crop using a glyphosate oxidoreductase or a glycine oxidase gene, it would not be exempt and the SECURE rule would apply because the crop-MOA combination being used has not been previously deregulated by APHIS.

In addition, APHIS exempted from its SECURE rule those genetically engineered organisms that APHIS has already determined are not and do not pose risks of being plant pests. Three clusters fit this exemption: (1) those genetically engineered plants that have successfully been granted non-regulated status through the petition process of the earlier APHIS regulations; (2) those genetically engineered events that have successfully passed through the previous “Am I Regulated?” process; and (3) those genetically engineered crops that pass through the initial review (consultation) process of the APHIS SECURE rule and APHIS determined that there is not a plausible plant pest risk.

In light of the first exemption, genetically engineered plant-trait-MOA combinations already deregulated continued to be deregulated and need not be reevaluated under the SECURE rule. In light of the second exemption, genetically engineered events that APHIS determined were not subject to regulation under the AIR process also remain outside the SECURE rule, though the exemption only applies to those plants specifically listed in AIR and not subsequent modified plants. APHIS also ended the AIR process and all genetically engineered plants in the future must follow the SECURE rule.

For those genetically engineered plants that are not within the exemptions or that have not already passed through an APHIS deregulation process, the SECURE rule created a regulatory status review (RSR). In accord with RSR procedures, developers of genetically engineered plants must submit required information for RSR. APHIS will conduct an initial review (consultation) to determine “whether there is a plausible pathway by which the genetically engineered plant … would pose an increased plant pest risk.” This APHIS review will focus on the MOA of the introduced genetic material in a given crop and will not require field trial data. APHIS will complete the evaluation for the plausible pathway within 180 days. If APHIS does not identify a plausible pathway, APHIS will post the plant, trait, and general description of the MOA on its website indicating that it is not subject to the regulation. If APHIS does find a plausible plant pest risk, the developer of a genetically engineered plant has three options: (1) seek a second, deeper status review and gain an APHIS determination that the genetically engineered plant is unlikely to pose an increased plant pest risk; (2) seek a permit setting forth the conditions for movement or use; or (3) withdraw the request. APHIS will also post on its website the plant, trait, and MOA of these genetically engineered plants initially found to have a “plausible pathway” to becoming a plant pest risk. APHIS will complete this more detailed RSR process within 15 months.

Comparing the APHIS RSR to the previous APHIS regulations, if in the RSR initial review (to determine if there is a “plausible pathway” to becoming a plant pest risk) APHIS finds a “plausible pathway” of a plant pest risk, the RSR process becomes equivalent to the permit and deregulation processes and procedures of the previous regulations. APHIS set the effective date for implementation of certain components of the SECURE rule as August 2020, with most provisions operational by April 2021, and fully operational in October 2021.

In comments accompanying the SECURE rule, APHIS stated plant breeders can make self-determinations that a particular genetically engineered plant-MOA combination is the same as that of a previously not regulated or deregulated genetically engineered plant. If a plant breeder is uncertain of this self-determination, or simply cautious about a particular plant’s regulatory status, APHIS allows plant breeders to seek confirmation of their self-determination conclusion; this is similar to the discontinued AIR process.^2^ The National Environmental Policy Act (NEPA) requirements apply for the SECURE rule. NEPA requires a federal agency to assess the environmental effects of their proposed actions (e.g., an environmental assessment or environmental impact statement) prior to making decisions.

#### Environmental protection agency (EPA)

The EPA exercises regulatory control over biotechnology through three statutes: the Federal Insecticide, Fungicide and Rodenticide Act (FIFRA), Section 408 of the Federal Food, Drug and Cosmetic Act (FFDCA), and the Toxic Substances Control Act (TSCA). The assessment of genetically engineered crops under FIFRA focuses on the pesticidal property rather than the crop itself. EPA obtains its authority to regulate pesticides from FIFRA, including substances that plants produce for protection against pests, known as plant-incorporated protectants (PIPs), which includes some plants created via biotechnology, such as crops containing rDNA coding for expression of *Bacillus thuringiensis* (Bt) insecticidal proteins or edited for pest resistance. The specific technology used to modify the DNA of a plant is not the relevant criterion in determining whether the substances comprise a PIP. Rather, EPA advises that the intended use and claims made for preventing, destroying, repelling, or mitigating a pest determine whether that particular use is pesticidal. Thus, EPA regulation of PIPs is not based on the specific biotechnology used to modify the plant or the nature of the modification.

In October 2020, EPA proposed a new rule that would exempt certain PIPs from registration requirements and from the requirement of establishing a tolerance exemption. The proposal has not been finalized at the time of this writing; there is an ongoing public comment period (EPA 2020). Similar to the underlying principles of the SECURE rule, PIPs consisting of deletions created through GEd and that result in the reduction or elimination of a substance, and other PIPs that are found in sexually compatible plants would be exempted as long as they “pose no greater risk than PIPs that meet EPA safety requirements,” and “could have otherwise been created through conventional breeding”.

Under the proposed rule, a PIP may be exempt when the pesticidal substance is identical to a substance found within a plant or its sexually compatible relatives or when no protein is produced. It is proposed that silent mutations would be allowed in the DNA sequence, as long as they do not alter the amino acid sequence of the product. When using templated GEd technology, the insertion must be into intergenic space. Furthermore:

- The expression of the pesticide in the new variety of plant cannot exceed the variable expression in the sexually compatible plants. Thus, the exemption does not apply if the developer develops a stronger pest dosage than available in the sexually compatible plant.
- The expression of the pesticide must not create new exposures to humans or the environment (e.g. other insects) than what already exists in the sexually compatible plants.
- The expression of the pesticide in the new plant must be in the same tissues and the same developmental stage as the pesticide trait in the sexually compatible plant. For example, developers are not allowed to move a root tissue pesticide trait from a sexually compatible plant to the leaves of the new plant.

Finally, EPA is proposing a requirement for developers to submit a letter of self-determination or to request EPA confirmation that a PIP based on a sexually compatible plant created through biotechnology meets the criteria for exemption set forth in the new proposed rule. If the new PIP is not within the exemption, a registration under FIFRA is required.

#### Food and Drug Administration (FDA)

The FDA has regulatory control over food from plants developed via biotechnology and all forms of genetic modification, including conventional breeding, under the Federal Food, Drug and Cosmetic Act (FDCA). FDA has a voluntary consultation process for foods derived from biotechnology (FDA 1992), whereby producers of genetically engineered crops and ingredients voluntarily consult with FDA about their genetically engineered crops prior to commercial release.

“Voluntary consultation: Sellers have the obligation to ensure that the food and feed they sell is safe and legal for human and animal consumption, regardless of the method or technology used to produce the food or feed. While FDA has no premarket authority over whole foods (as in contrast with food and color additives), it has power to take enforcement action against food that is not safe or legal. Hence, if a seller has any doubt over the safety of their product, they should consult with FDA. In addition, buyers, shippers, or traders of food and feed products may require FDA consultation before agreeing to buy or ship a product.

In January 2017, FDA requested comments and responses to specific questions about GEd in new plant varieties used for food (FDA 2017). FDA sought comments on the relevance of FDA’s experience under the 1992 consultation process for GEd crops and whether a scientific basis existed for concluding that GEd crops were unlikely to present food safety risks different from those of crops developed through traditional plant breeding. In 2018, FDA released its “Plant and Animal Biotechnology Innovation Plan,” indicating plans to clarify its policy approach to food safety evaluations for GEd crops by developing guidance, with specific references to foods produced using GEd crops (FDA 2018). FDA continues to accept voluntary consultations for food from GEd crops in the same manner as has been done with genetically engineered crops since 1992.

#### Disclosure or labeling of foods containing bioengineered content

In December 2018, the USDA-Agricultural Marketing Service (AMS) released the National Bioengineered Food Disclosure Standard (NBFDS) (USDA-AMS 2018). USDA-AMS is a marketing division of USDA and they have stressed that disclosure standard is for purposes of consumer information and does not say or imply, explicitly or implicitly, anything about the nutrition, safety, or environmental attributes of the disclosed food.

While the NBFDS regulations, did not specifically address whether foods containing products developed using GEd techniques require “bioengineered” disclosure, the scope of disclosure only covers changes made by rDNA and that cannot be created through “conventional breeding or found in nature.” Those foods containing products created using GEd that do not create novel DNA combinations that could not be created by “conventional breeding or found in nature” would not require disclosure (AMS BE disclosure website).

### Africa

#### Introduction

GEd holds huge potential benefit as it allows the relatively quick, efficient, accurate and cost-effective modification of valuable genetic traits in crops, livestock and micro-organisms. Particularly under-resourced research as well as development and innovation environments, such as those in the public sector and developing countries, stand to benefit from it. Using it in combination with established breeding programs could effectively decentralize more sophisticated genetic improvement capabilities to allow a wider variety of innovators to deliver valuable, locally-relevant cultivars/breeds (Whelan et al. 2020). In the African context this could not only contribute directly towards local and regional food security, but also serve as a bioeconomy-based springboard for sustainable regional development.

To realize these benefits, fit-for-purpose governance frameworks that satisfactorily manage national priorities and allow meaningful regional integration, must be established in each country. However, GEd is inextricably linked to GE and GMOs—also in the minds of broader society where these technologies, particularly when applied in foods, are contentious and divisive issues. The establishment of appropriate governance frameworks is therefore no longer the relatively simple, science-dominated exercise it was in the 1970s, but one informed by a multitude of diverse and context-specific perspectives and issues.

Unsurprisingly, discussions regarding the governance of GEd therefore invariably raises questions whether the organisms resulting from its application should be considered “genetically modified” or not—and as a result be regulated as such or not. However, genetic engineering technologies have evolved to a point where many of the original assumptions, on which current GMO governance frameworks were based, are no longer valid. Trying to treat GEd and conventional breeding techniques as completely divergent approaches with distinct risk profiles, based on the interpretation of outdated legal definitions, therefore establishes a false dichotomy (NASEM 2016). Care should be taken to prevent such non-discriminating, inaccurate risk management conclusions from unnecessarily delaying the establishment of fit-for-purpose governance frameworks and the sustainable application of the relevant technologies.

The status of discussions and developments regarding GEd governance across African countries is diverse and generally influenced by

(i) The status of the national GMO regulatory framework, including the National Biosafety Authority’s (NBA’s) experience with GMO regulation and consequent decision-making confidence. In environments with little GMO experience, GEd is sometimes perceived as a “further development of GM-technology”, purely based on the chronology of these technology developments and “placed on a back burner until GMO issues can be resolved”.
(ii) The national appetite and capacity for genetic modification research, development and innovation. National capacities in advanced genetic improvement technologies remain low across many African countries and associated products are often still perceived as not having much local relevance based on the few GM crops/traits that have been approved elsewhere. A country’s research and development capacity also has a direct impact on the performance of the regulatory system, which in almost all cases depends on the local research and development fraternity for the relevant technical risk analysis expertise.
(iii) The perceived levels of public support. “Public license” has grown to become a critical prerequisite to genetics-based innovation. Unfortunately, *perceptions* regarding public acceptance and associated political will are often strongly influenced by local interest groups. The limited available scientific data on the acceptance of agricultural biotechnology in Africa invariably highlights the inaccuracy of these politicized perceptions (Gastrow et al. 2018).
(iv) The conflation of different genetic modification technologies, genetic impacts, applications and risk profiles. GEd discussions are often negatively impacted by an inability to separate genetically engineered products/applications with very distinct risk profiles, e.g. equating GEd with gene drives. The convoluted discussions on synthetic biology under the Cartagena Protocol on Biosafety (CPB) has exacerbated rather than alleviated this problem.

#### Status of genome editing regulatory discussions across Africa

Kenya and Nigeria have led the continent in establishing formal guidelines on how to incorporate GEd and other technologies into their regulatory frameworks.

**Kenya** is finalizing its first draft of their new guidelines but has already indicated that a key provision will be the submission of a formal inquiry, to the Kenya National Biosafety Authority (NBA), to determine whether a proposed project falls within the mandate of the Biosafety Act’s regulations. These decisions will be made on a case-by-case basis and are likely to be based on the presence or absence of transgenic sequences, similar to the framework introduced by Argentina in 2015 (Whelan and Lema 2015). While these guidelines are still under development all GEd projects are subject to the Biosafety Act. To date the Kenya NBA has approved five (5) GEd projects for contained use activities (http://ke.biosafetyclearinghouse.net/approvedgmo.shtml, accessed 29 September 2020). These include three projects on plants to introduce disease resistance in banana and yam, as well as nutritional and agronomic enhancement of grass pea.

**Nigeria** recently became the first African country to publish a draft of their “*National biosafety guidelines for the regulation of gene editing*” (July 2020). This follows the amendment of the National Biosafety Management Agency (NBMA) Act in 2019 to include a section [25(A)] that states “*No person, institution or body shall carryout gene drive, gene editing and synthetic biology except with the approval of the Agency*”. The approach followed in the draft guidelines aligns well with those published for Argentina by Whelan and Lema (2015).

The most important aspects of the Nigerian guidelines can be summarized as follows:

(i) It includes clear reference and alignment with the provisions of the CPB.
(ii) It establishes a preliminary-consultation process to determine if a project and its resulting products will fall within the mandate of the NBMA Act.
(iii) The Act’s mandate is defined by the presence of “*a new combination of genetic material, e.g. uses a transgene which remains in the final product*”, i.e. a product-based interpretation.
(iv) The guidelines promote overall efficiency, accountability and transparency in applying ALL relevant regulations—suggesting an oversight and collaborative approach with other regulatory authorities, e.g. “*existing national legislation regarding conventional breeding or natural selection*”, in cases where the NBMA Act may have no jurisdiction.
(v) It includes clear administrative and contextual guidance, including in the form of a standard application form.
(vi) The Nigerian guidelines align well with the great majority of other international guidelines for plant-based GEd that have been published to date, including those of Argentina, Australia, Brazil, Chile, Colombia, India, Israel, Japan and Paraguay.

Although **South Africa** has the longest track record of GMO regulation and commercial use on the continent it has been slow to accommodate GEd requirements in its governance frameworks. The Academy of Sciences of South Africa (ASSAf), under the auspices of the Department of Science and Innovation (DSI), published a consensus study on “*The Regulatory Implications of New Breeding Techniques (NBTs)*” in March 2017 (ASSAf 2017), but no formal guidelines have been published yet.

The purpose of the ASSAf consensus study was to evaluate the risk/benefit implications of NBTs (including GEd), ascertain the applicability of existing legislation and possibilities for alignment in context of available international examples, assess the robustness of the current South African regulatory framework and risk analysis practices to accommodate these and future, related technologies and, finally, to make pertinent recommendation based on these findings.

The study’s findings and recommendations:

(i) NBTs hold great potential, particularly for developing biotech innovation systems and are therefore relevant to South Africa.
(ii) Only a few countries have (had) formalized NBT regulations (as of March 2017 when the study was published), however a clear risk analysis-based consensus was emerging from international science-based discussions.
(iii) Genome modified (including GEd) organisms are the principal source of risk. These *products* should therefore be the trigger and subject of regulation.
(iv) South Africa’s GMO Act provides an adequate framework for NBT regulation and based on the definition of a GMO therein, i.e. *“…an organism the genes or genetic material of which have been modified in a way that does not occur naturally*…”, the threshold for regulation is genetic variation beyond that which may occur naturally.
(v) A succinct, case-by-case consultation process should be established under the GMO Act to determine if a product should be regulated as a GMO or not (similar to the then recently published Argentine framework).
(vi) The likely regulatory outcomes of the suggested framework align well with the (then) current consensus risk analysis discussions.

Even though the ASSAf consensus study used and recommended a risk analysis (science-based) approach to suggest risk-appropriate GEd regulations/guidelines, subsequent discussions of the regulatory authorities apparently got bogged down in legal interpretations of the existing legislation. The central question evidently being whether the GMO Act has a “product”, “process” or dual basis—a reflection of an enduring international debate which is further discussed below. Moreover, South Africa’s continued use of the European-based definition for GMO has also been widely questioned, primarily because of its divergence from the Cartagena Protocol’s definition (to which South Africa is a party) and use of the value-laden, capricious concept of “*naturally occurring genetic variation*” as a threshold for GMO regulation (see Tagliabue 2016 for a European-focused discussion on this topic). Although not ideal, it could however be argued that when interpreted from a product perspective “*genetic change that does not occur naturally*” could have the same meaning and implications as “*novel combination of genetic material*”, allowing the conceptual alignment of the South African framework with the current consensus international approach to GEd regulation.

Several South African laboratories are engaged in contained GEd research and development work involving model plants like tobacco and *Arabidopsis*, diverse crops like *Eucalyptus* and grapevine, as well as micro-organisms. All this work is done in registered GMO facilities because recombinant DNA technology and intermediary GMOs are an inherent part of the development process for GEd organisms. No application which involves activities with an organism with only genome edits have to date been considered by the Executive Council of the GMO Act.

Other African countries that have GMO governance frameworks and have “started to consider GEd guidelines” include Burkina Faso, eSwatini, Ethiopia, Ghana, Sudan, and Zimbabwe.

#### Context-relevant discussion and recommendation

Genome editing governance guidelines are as a rule developed as amendments to GMO regulations, making the existing regulations a defining departure point. When the legal interpretation of these, often technically outdated frameworks, take precedence over the science-based principle of sound risk management, the establishment of new fit-for-purpose guidelines is often impeded. A prime example of this is the debate on the questionable distinction between “product” and “process” based regulatory systems.

This possible distinction was not a critical consideration when the definitions possible risks, and resulting regulatory frameworks for, GMOs were first contemplated in the 1970s to 1990s. In context of the then predominantly science-based approach to risk management/regulation, it was clear that possible risks could only stem from the organism (product) itself, mediated through the GM phenotype, and the possible genetic outcomes of the limited available technologies (processes), resulted in an apparent clear dichotomy (see McHughen 2016 for an overview). In addition, this distinction has little significance from a science-based risk analysis perspective, because it considers both, as relevant, on a case-by-case basis for all regulated entities. It could, however, have a significant impact on the scope of regulation as it defines divergent triggers for regulation. As genetic engineering technologies continued to evolve, the diversity of possible genetic outcomes increased to a point where their inclusion or exclusion from GMO-specific regulations could depend on the interpretation and application of a “process” or “product” regulatory trigger.^3^ The general debate on “product-” versus “process-based” regulatory triggers, both in terms of its application to current and possible amended, future regulatory frameworks, has therefore intensified over the past 10 years as the diversity of genetic engineering techniques and possible genetic outcomes evolved..

A key assumption of a process-based regulatory approach is that products resulting from the regulated process are all fundamentally different, riskier and therefore in need of formal risk assessment and management. In the GMO context, it therefore tends to increase the scope of regulation. In general, it is therefore favored by those who want to limit the use of the technology. In contrast, those who promote the sustainable use of the technology argue that only product-based regulation makes sense from a scientific, risk management perspective. Firstly, because the principal source of risk is the features of the modified organism itself (the product), and not the technique/process through which it was generated. Secondly, the process through which genetic variation is induced is not an accurate determinant of the ultimate characteristics of the resulting products and will therefore create scientific, legal and/or administrative incoherence.

These new technological developments and consequent need for updated governance guidelines should be used as an opportunity to realign regulatory frameworks with scientific risk analysis principles. Despite the enduring public debate on genetics-based innovations there is wide consensus among the scientific community that these technologies can be applied in a useful and safe manner. The well-established science-based risk analysis frameworks for GMOs have evolved over more than three decades into robust tools that can be effectively applied to any genetically engineered organism, to ensure its sustainability, if the principle of a case-by-case, comparative risk analysis is applied (Duensing et al. 2018). We therefore have an opportunity not only to address the shortcomings of past regulations, but also to future-proof them—something which is possible if regulations are based on the well-established, scientific risk analysis principles.

Broadly speaking the current regulatory approaches followed by several countries interested in the responsible application of GEd, including the Nigeria example above, align well and represent a viable and responsible option when

(i) a novel combination of genetic material (equivalent to “*modified in a way that does not occur naturally*” in the South African and EU definitions of GMOs when interpreted from a product perspective) is used as the discriminator between GM and non-GM regulation—offering the opportunity to exclude relevant genotypes from GMO regulation and bias, and
(ii) oversight is handled on a case-by-case basis to allow an acceptable level of flexibility as technologies and products evolve, while ensuring good governance.

### Australia and New Zealand

#### Introduction

The regulatory requirements for GEd in Australia and New Zealand are intrinsically linked to the regulation of GMOs. The structure of the regulatory systems provides insight into the potential pathways to market for GEd products.

In Australia, GMOs, including GEd plants, are regulated by a range of agencies with the appropriate expertise to assess any risks that may be associated with the GM product (Thygesen 2019; Table 1 of Supplemental Information).

The regulatory scheme is underpinned by the *Gene Technology Act 2000* (Cth) (GT Act, Commonwealth of Australia 2000), the *Gene Technology Regulations 2001* (Cth) (the GT Regulations, Commonwealth of Australia 2001), and corresponding state and territory legislation. The GT Act regulates the process of ‘gene technology’ rather than the products themselves (*cf* Canadian novel foods regulations, Ellens et al. 2019) using a risk analysis framework that provides consistent and rigorous risk analysis to regulated activities (OGTR 2013).The breadth of processes and outputs/products that may be covered by the Act is vast and requires case by case assessment including GEd.

Under the national scheme, all states and territories recognize approvals of GMOs made by the Regulator with respect to potential harm to human health and safety and the environment. However, under an intergovernmental agreement, states and territories reserved the ability to legislate with respect to market and trade. In 2003 and 2004, various bans on the commercial cultivation of GM crops, or more specifically GM canola varieties, were implemented by most state governments in Australia. Various subsequent reviews of state legislation, driven by strong advocacy from the grains industry (e.g. Single Vision Grains 2007) have subsequently led to wider commercial GM canola production. The current status of moratoria in Australia are summarized in Table 2 of the Supplemental Information.

In most states, legislation remains in place that provides a mechanism to ‘block’ commercial production of plant and/or food products from gene technology on a market and trade basis. Where an order is in place, applicants may seek an exemption order allowing for certain activities on a case-by-case basis. However, the legislation is seen by industry as restrictive and a potential barrier to commercialization.

In New Zealand, GMOs, including GEd products are primarily regulated under the *Hazardous Substances and New Organisms Act 1996* (the HSNO Act, New Zealand Government 1996) and administered by the Environmental Protection Authority (EPA). Under the HSNO Act, a ‘new organism’ includes an organism that was not present in New Zealand before the 29th July 1998 and a GMO. The main laws governing GMOs in New Zealand are listed in Box 1 of the Supplemental Information.

New Zealand has signed and ratified the Cartagena Protocol for Biosafety and as such adopts a precautionary approach to GMOs. The Ministry of Foreign Affairs and Trade have overall responsibility for functions pursuant to the Cartagena Protocol on Biosafety and is supported by the EPA, the Ministry for Primary Industries (MPI) and Food Standards Australia New Zealand (FSANZ). Importantly, EPA, MPI and FSANZ all operate cost recovery models for the assessment of applications and the monitoring and enforcement of approvals.

New Zealand Government policy on GMOs is guided by the findings in a report issued by the Royal Commission on Genetic Modification (2001). Accordingly, the EPA must consider the potential effects of a GM product on the environment, health, and safety of people, the economy, the social and cultural well-being of people and communities, Māori culture and their relationship with the environment, as well as international obligations (e.g. The Codex Alimentarius, CAC 2009).

The New Zealand regulatory system is unique in that there is a requirement to consider the costs, benefits and potential risks of an application. The system considers that where the benefit of a ‘new organism’ sufficiently outweighs the potential harm then that product should be allowed.

A major consideration for the EPA in assessment of applications includes ensuring Māori are engaged in its activities and decision-making processes. This ensures that the regulatory process reflects the concept of partnership between the government and the indigenous people as implied in the Treaty of Waitangi of 1840 (New Zealand Ministry of Culture and Heritage 2020).

Anyone planning to submit an application or proposal to the EPA must engage with Māori groups whose interests could be affected by the application.

#### A bi-national food regulation system

A cooperative bi-national arrangement involving the Australian Government, states and territories and New Zealand establishes the food regulation system for both countries (Kelly 2019). Food Standards Australia New Zealand (FSANZ) is an independent statutory authority with responsibility for developing food standards that protect public health and safety, providing adequate information and preventing misleading conduct.

The Australia New Zealand Food Standards Code (the Code) is a collection of enforceable food standards. Both Australia and New Zealand food laws provide that it is an offense to supply food that does not comply with the Code. Any agency, body or person can make an application to vary the Code.

In contrast to both OGTR and EPA, FSANZ assesses the final product for safety of food derived from gene technology rather than the process, albeit the assessment looks at the process of product development. Further, the definitions that FSANZ are guided by differ from those of the OGTR and EPA, but are broad enough to consider products of GEd.

The sale of food produced using gene technology in Australia or New Zealand is illegal unless expressly permitted. All such foods intended for sale must undergo a pre-market assessment under Standard 1.5.2—Food Produced Using Gene Technology contained in the Code. In some circumstances, proponents may also be required to submit an application to amend Standard 1.5.1 Novel Foods. The Standards have two provisions—mandatory pre-market approval (including a food safety assessment) and mandatory labelling requirements. The Standards ensure that only assessed and approved foods derived from gene technology enter the food supply.

If the OGTR and or the EPA determine an edited product has been developed using gene technology and requires regulation, then typically FSANZ will also need to consider whether a change to the Code is required. In many cases the data requirements provided to each of the competent authorities are similar. Importantly, whilst there is no specific animal feed approval, GM crops grown for feed cannot be grown in Australia or New Zealand unless it has been approved for human consumption.

#### Consideration of new breeding technologies (NBTs) in Australia and New Zealand

Consideration of new breeding technologies (NBTs) has been on the Australian and New Zealand regulatory agenda since 2012. At that time, the New Zealand EPA was asked whether organisms created using zinc finger nuclease 1 (ZFN-1) and transcription activator-like effectors (TALE) are GMOs and therefore subject to the HSNO Act (Kershen 2015). The request was made under a special procedure (‘Determination’) as set out in Section 26 of the HSNO Act for defining whether something is a ‘new organism’. The EPA convened a panel to assess the determination and in 2013 concluded:

1. That ZFN-1 and TALE organisms do meet the definition of a GMO; but
2. Are ‘similar to’ a technique excluded from the Act under regulations.

As a result, the panel resolved that organisms altered through the use of ZFN-1 and TALE are not GMOs. However, in 2014 this administrative decision was challenged in the High Court, which ruled that the EPA did not have the authority to make such a decision since it was a legislative matter. As such, in New Zealand, all GEd techniques remain subject to regulation under the HSNO Act as GMOs.

Around the same time, FSANZ consulted with experts and sought scientific views on whether foods derived from plants developed using NBTs should be regarded as GM food, or whether they are more like conventional food. Participants from a 2013 workshop (FSANZ 2014) concluded that there are basically 3 non risk-based categories that could determine the regulatory requirements of products derived from gene technology:

- Category 1: Comprises cisgenesis, intragenesis, some uses of Site Directed Nucleases (SDN) and GM rootstock grafting. Products derived from these techniques would be regarded as GM, although a simplified form of safety assessment may be warranted
- Category 2: Includes Oligo Directed Mutagenesis (ODM) and some uses of SDN, where products derived from them would not be regarded as GM
- Category 3: Comprises gene technologies at an early stage that are separated from the final product during the breeding process, such as reverse breeding. For products in this category, the panel concluded that they are not GM, but there is a need to confirm the reliability of the breed out process.

In 2018, FSANZ began further consultation with key stakeholders and the community to look at how food derived from NBTs should be captured for pre-market approval under Standard 1.5.2 and whether the definitions for *'food produced using gene technology'* and *'gene technology'* in Standard 1.1.2–2 should be changed to improve clarity about which foods require pre-market approval (FSANZ 2018; Kelly 2019).

FSANZ established an Expert Advisory Group (EAG) to provide advice on issues relevant to the review. Advice from the EAG and key stakeholders resulted in a 2019 report (FSANZ 2019) that made three recommendations:

1. FSANZ prepare a proposal to revise and modernize the definitions in the Code to make them clearer and better able to accommodate existing and emerging genetic technologies.
2. As part of the proposal, FSANZ give consideration to process and non-process-based definitions and the need to ensure that NBT foods are regulated in a manner that is commensurate with the risk they pose.
3. Throughout the proposal process FSANZ will ensure there is open communication and active engagement with all interested parties and also explore ways to raise awareness about GM and NBT foods.

The next steps will consider an amendment to the definitions in the Code and this has been added to the current FSANZ Standards Work Plan.

In October 2016, the OGTR released a discussion paper ‘Options for regulating new technologies’ under a technical review of the GT Regulations (OGTR 2016; Thygesen 2019). The primary aim of the review was to provide clarity about whether organisms developed using new technologies are subject to regulation as GMOs and ensure that new technologies are regulated in a manner commensurate with the risks they pose.

The separation of policy and regulation is a standard governance arrangement in place for most regulatory agencies of the Australian Government. As such, the Regulator’s technical review could not alter the policy settings of the Scheme. Therefore, the technical review was limited to only consider:

- cases where the capture or exclusion of these techniques is not clear, and whether those new technologies should be regulated, and
- scientific evidence relating to risks posed as a result of using new technologies.

The discussion paper canvased four broad options for how clarity about regulation of specific new technologies could be achieved. The Regulator sought submissions from interested parties on the merits of these options, in particular in response to a set of consultation questions.

After extensive consultation, outcomes of the technical review (OGTR 2019) were ratified by government, approving a set of amendments to the GT Regulations. Those amendments are progressively being introduced.

The key amendment that came into effect from the 8th October 2019 means that any product modified using SDN and allowing DNA repair via Non-Homologous End Joining (NHEJ), are no longer considered a GMO under the regulations. However, if a template is provided to guide repair via homologous recombination, then the product would be considered a GMO and be subject to regulation. Similarly, any new technologies that use other approaches or enzyme systems (e.g. ODM, nickases, Prime editing etc.) would also be deemed a GMO and therefore be regulated.

These changes are at incongruent with the science-risk based system and bear no relation to the potential harm posed by or as a result of the different GEd processes. Rather the distinction is based on what the Regulator could achieve under the limited scope of the review and the challenges of where to draw the line on the extent of changes that could be made with the assistance of template guided repair. Such changes require amendments to the policy setting of the Scheme.

A review of the regulatory scheme has also been conducted to look at the policy framework for the regulation of gene technology with a final report issued in October 2018 (Commonwealth of Australia 2018). Of the 27 recommendations, only a few have direct relevance to NBTs. Specifically:

- the ability to capture a broader scope of activities within the Scheme, via the process trigger, should be maintained. This means that products derived from NBTs will continue to be considered GMOs
- the incorporation of a principles-based approach to some aspects of regulation would facilitate better alignment of regulation to the level of risk. This will perhaps allow the Regulator the ability to, in the future, apply a ‘soft’ touch to low risk products derived from NBTs.

The review implementation action plan has a 5-year timeframe with priority given to progressing recommendations relating to definitional considerations and the development of additional risk tiering. However, it is unclear on the timeframes for any substantial amendments to the Act that could facilitate a pathway to market for GEd plants.

#### Challenges and opportunities

The OGTR regularly commission a survey to gauge Australian community attitudes to gene technology. A consistent feature of these surveys is that consumer support for GM technologies is largely conditional, but there is a high level of trust in the Regulator and the regulatory system. GEd products with the right traits may be a catalyst for growing this support. Findings from the most recent survey suggest that there is significant awareness and acceptance of GEd with many respondents considering that it might improve our way of life (Cormick and Mercer 2019).

Despite the differences discussed above, the regulatory systems of both Australia and New Zealand are well respected internationally. In particular, many companies exporting biotech-based products will seek approval from FSANZ and other jurisdictions prior to going to market. The FSANZ approval is seen as an integral part of good product stewardship and an insurance against adventitious presence issues with respect to trade. Further, whilst a FSANZ assessment attracts a cost recovery fee, a desirable feature of the system is that the assessment process is time bound. Therefore, for a commercial company there is an element of certainty around regulatory approval. This contrasts with many other systems around the world where the timeframes for assessment are not defined (e.g. Canada and China).

The recent changes to the Australian *Gene Technology Regulations* consider GEd products derived from NHEJ as non-GM. This offers significant opportunity for developers to include simple editing as part of their breeding programs. However, developers need to be aware that until such time as the editing process is completed, the dealings may still be considered a GMO and they will need to adhere to the relevant regulations no matter how arbitrary, capricious, and detrimental to the public welfare they may be. Further, once an edited product has been confirmed, it could be cultivated in Australia but may not yet be able to enter the food chain without a food safety assessment and amendment to the Food Standards Code through FSANZ. There is currently regulatory asynchrony in Australia between OGTR and FSANZ and until such time as FSANZ complete its current review this will continue to create uncertainty.

It is yet to be determined what role the states and territories will play in the commercial pathway to market for GEd food products in Australia. Existing legislation offer opportunities to ban products on the basis of potential or perceived impacts on market and trade and this could be influenced by public/political sentiment. This was seen with the introduction of GM canola and a potential barrier to commercialization.

GMOs continue to be a politically sensitive subject in Australia and New Zealand with strong vocal opposition from minority political parties and anti-GM non-governmental organizations. These groups seek to prevent commercial release of GM products as well as impose restrictions on the consumption of foods with GM content. The main driver for opposition is concern that GM products may tarnish the clean/green image of Australia and New Zealand and negatively impact domestic and export price premiums to some markets. Despite these barriers, many researchers, primary producers and industry groups remain supportive of GEd technologies and continue to undertake research and development towards product commercialization.

### Japan

#### Introduction

Japan remains one of the world’s largest per-capita importers of food and feed products produced using modern biotechnologies. As a key purchaser of (GEd food and feed products, the Japanese government's regulatory approach to GEd food and feed products is important to global food and feed production and distribution and is receiving worldwide attention. Throughout 2019 and early 2020, Japanese regulators completed the handling guidelines for GEd food and agricultural products. These guidelines provide the commercialization pathway for developers who wish to commercialize their products in Japan. The Ministry of Health, Labor and Welfare (MHLW) and the Ministry of Agriculture, Forestry, and Fisheries (MAFF) convened committees of technical experts to provide guidance throughout development of the guidelines, held public comment periods, and published their respective guidelines for GEd food and agricultural products. Researchers in Japan have developed a few GEd crops such as tomato, potato, rice etc., but none are as yet commercially available. Encouragingly, however, after discussions with the regulatory authorities, notification for marketing GEd tomatoes was granted in December 2020. There is limited applied research and development of animal biotechnology in Japan, and most activities remain in the area of basic research. Researchers are developing a GEd red seabream and blowfish, but they are not yet commercially available.

#### Regulatory status of GEd organisms in Japan

Details of the history and current status of regulation of GEd organisms based on the Cartagena Law of Japan are summarized in the paper “Regulation Status of Genome-Editing Organisms Based on the Cartagena Law of Japan” (Tsuda et al. 2019). Here is a summary of the current situation.

In Japan, a developer of GMOs is required to receive three different approvals on food, feed, and environmental safety (that is, the impact on biodiversity) prior to commercial distribution of the products in Japan under the Food Sanitation Act (Ministry of Justice 2020a), the Feed Safety Act (FAMIC 2020), and the Cartagena Act, respectively (Ministry of Justice 2020b). Regulation of genetic modification in Japan is governed by three ministries—namely, the Ministry of the Environment (MOE), the Ministry of Agriculture, Forestry and Fisheries (MAFF), and the Ministry of Health, Labor and Welfare (MHLW).

These regulatory agencies developed necessary policies and procedures for handling GEd products falling within their purview. In 2016, at the Expert Committee on LMOs of the Nature Conservation Committee, the Central Environment Council, the MOE issued a report entitled “Examining enforcement of the Cartagena Act,” which pronounced decision-making on the regulatory status of organisms that do not contain exogenous nucleic acids created by new breeding techniques such as GEd as an urgent issue and stressed the necessity of carefully considering this status in light of the latest scientific knowledge and international harmonization (MOE 2018a). In July, 2018, the Expert Meeting on Genome Editing Technologies under the Cartagena Act was established within the Expert Committee on LMOs of the Nature Conservation Committee, the Central Environment Council, the MOE, as the administration of the Cartagena Act (MOE 2018b). After multiple discussions in the Expert Meeting, a draft report entitled “Classification and status of organisms produced by application of GEd technology under the Cartagena Act” (MOE 2018c) was finalized by the Expert Committee. The draft report was discussed at the Nature Conservation Committee and Central Environmental Council (MOE 2019a) after one-month public comment period, and in February, 2019, the MOE reported the final decision (MOE 2019b).

GEd techniques are classified into three principal categories: site-directed nuclease (SDN)-1—that is, site-directed mutagenesis; SDN-2—that is, templated editing; and SDN-3—that is, site-directed gene insertion. Three types of artificial nucleases used for targeted modification are considered: zinc finger nucleases; transcription activator-like effector nucleases; and CRISPR/Cas9.

Because the end-products of the SDN-1 methods do not contain inserted nucleic acid or its replicated product, they do not meet the definition of LMOs in the Cartagena Act. On the other hand, the end-products of the SDN-2 and SDN-3 methods might contain inserted nucleic acids processed extracellularly, and are therefore categorized as LMOs. This categorization is the same as that in a document issued by the Australian Government. The size of the nucleic acid insert is undefined in the Cartagena Act. Any organism with inserted extracellularly processed nucleic acid (including RNA) is regarded as an LMO, and is automatically subject to the regulations stipulated in the Cartagena Act unless the complete removal of the inserted nucleic acid (including RNA) or its replicated product is confirmed. The final determination according to the MOE approach would be applicable to null segregants, from which the inserted foreign gene has segregated. Even if the products are developed by SDN-2 and/or SDN-3 methods, these products would be exempted from LMO regulations if these are applicable to self-cloning or natural occurrence under the Cartagena Act.

The newly developed biotechnological end-products have to be rigorously classified in terms of whether they do or do not contain extracellularly processed nucleic acids. Developer or users are requested to notify the government with information on end-products created through GEd technology, including a product description and any knowledge of their impact on biodiversity prior to use. The competent national authorities [administrative agencies such as the MAFF, the MOE, and the Ministry of Education, Culture, Sports, Science and Technology (MEXT)] call on users of GEd SDN-1-based technologies to report prior reviews of the biological characteristics and impact on biodiversity of GEd organisms to the appropriate ministry. In the case of a probable risk to biodiversity, the competent national authority will require additional information from the developer; thereafter, the necessary measures can be taken.

In response to the MOE's decision above, regulatory agencies developed necessary policies and procedures for handling GEd products falling within their purview. In October 2019, the MAFF’s Plant Products Safety Division published final guidelines on the “Specific Information Disclosure Procedures of Living Organisms Obtained through Use of Genome Editing Technology in Agriculture, Forestry and Fishery Fields” (USDA 2019b). In February 2020, the MAFF’s Animal Products Safety Division released the final guidelines for the handling of GEd feed and feed additives (USDA 2019c). In October 2019, the MHLW released the final guidelines for the handling of GEd food and food additives (USDA 2019d).

In Japan, product developers are requested to follow the relevant guidelines before commercializing GEd products. Developers should therefore consider addressing all three commercialization pathways for their product, depending on how it might be used in Japan.

The MAFF’s and MHLW’s guidelines are largely in alignment and both are claimed to be based on science. However, there are key differences in how the regulators determine whether a product is eligible for notification or must undergo the more significant safety review required of GMO products. Neither the MAFF nor the MHLW have specified how long developers should expect the consultation response or publication of information gathered through the notification process to take.

One of the most important requirements in the guideline to judge whether a product is eligible for notification or must undergo the more significant safety review required of genetically engineered products is to confirm removal of exogenous DNA integrated into the genome of an organism. Although the appropriate detection method is not legally determined in Japan, scientists have developed the k-mer method, a simple and high-throughput method for detecting exogenous DNA remaining in null segregant (Itoh et al. 2020).

The topic of “off-target” effects is one of the most discussed in the evaluation of GEd. The question is whether a similar approach should be considered across the assessment of off target risks for animals, plants and microorganisms in Japan. The presence or absence of off-target environmental risks is not covered by the LMO regulations, and in the case that the final product is likely to have an impact on the environment, more information is required. With regard to food safety, the MHLW requires that the presence of off-target mutations be checked using a search tool.

#### Domestic development

The first product to complete either the MHLW or MAFF ministry’s voluntary notification process for verification of whether a genome edited product should be regulated as a GE product (USDA 2020) is a. GABA-rich tomato. This GEd tomato expresses five times the normal amount of GABA, an amino acid linked to lower blood pressure, thanks to tweaks to genes that normally limit GABA production (Nonaka et al. 2017). On December 11, 2020, both ministries announced their determination that a genome edited tomato will NOT be regulated as a genetically engineered (GE) product.

The development of potatoes with reduced levels of glycoalkaloids is also in progress (Nakayasu et al. 2018). Although GEd has hardly advanced in Japan for livestock, improved breeds of fishes—especially those strongly preferred among Japanese consumers, such as Tai (Red seabream))—are being created (Ohama et al 2020). Only tomatoes are close to being assessed for safety. The GABA-rich tomato was developed in Japan, and it will be cultivated domestically and is being considered for domestic consumption. We think international import and export will be difficult unless the policy and procedures for handling GEd products are internationally harmonized. Nevertheless, it will be possible to grow them in countries where they are more readily permitted. The Japanese government is likely to approve the imports of crops like GABA rich tomato cultivars. As there are only a few examples of practical applications in Japan, it will be handled on a case-by-case basis in the future.

### Philippines

#### Introduction

Harnessing science and technology has been the Philippines’ stance as a country since the late nineties, as reflected by the Philippine Constitution of 1987 and the Republic Act 8435 (1997), primarily aimed to promote modernization of agriculture and fisheries. In line with this, the government formed the National Committee on Biosafety of the Philippines (NCBP) in 1990 through Executive Order No. 430 to develop a national strategy to form policies regarding biotechnology.

Following the Philippine government’s decision to become a signatory to the Cartagena Protocol on Biosafety to the United Nations Convention on Biological Diversity in 2000, the Department of Agriculture (DA) Administrative Order (A.O.) No. 08 or the “Rules and Regulations for the Importation and Release into the Environment of Plants and Plant Products Derived from the Use of Modern Biotechnology” was created in 2002. This was followed by the promulgation of Executive Order No. 514 was promulgated in 2006, which established the National Biosafety Framework (NBF); hence, solidifying the country’s commitment to the safe and responsible use of modern biotechnology and its products.

In 2016, consistent with the NBF development, the country established the Department of Science and Technology (DOST)-DA-Department of Environment and Natural Resources (DENR)-Department of Health (DOH)-Department of Interior and Local Government (DILG) Joint Department Circular (JDC) No. 1, which replaced the DA A.O. No. 08 (2002–2015). JDC No. 1 is also known as the “Rules and Regulations for the Research and Development, Handling and Use, Transboundary Movement, Release into the Environment, and Management of Genetically-Modified Plant and Plant Products Derived from the Use of Modern Biotechnology”. However, it is important to note that JDC No. 1, as well as the other existing national and local policies issued so far, are all about genetically modified organisms (GMOs), which characteristically have novel combinations of genetic materials as a result of the use of modern biotechnology.

#### Philippine policy on new plant breeding techniques (NBTs)

NBT or Plant Breeding Innovations (PBIs) is a new set of molecular, genomic and cellular tools that enable the targeted and efficient development of new varieties of crops with desired traits in a way that is faster and more precise than conventional plant breeding techniques, and may not result in novel combination of genetic materials.

To promote the responsible use of this technology and address the possible influx of products derived from these modern biotechnology tools into the country, the DA Biotechnology Program Office (DA-BPO) initiated a Study Group in early 2018 to look into the state of the art, regulatory landscape, applicable domestic laws and policies, and current capabilities of public R&D institutions on NBTs. The output of the said study group titled “A Review of the New Plant Breeding Techniques (NBTs) from the Viewpoint of Regulation” was forwarded in May 2019 to the NCBP, which subsequently created an Ad Hoc Technical Working Group in June 2019 that aimed to look into NBTs and assist in developing guidelines or amending existing biosafety guidelines to address issues unique to NBTs.

It is important to note that the Ad Hoc TWG has considered existing policies on NBTs developed in other countries in crafting its recommendations, which was submitted to NCBP on March 2020. As of now, the general policy or resolution on the regulation of plant and plant products derived from the use of NBTs has just been signed by the different agencies/offices comprising the NCBP for their approval, and will be released soon. The resolution covers eight different techniques which include the following: SDNs, ODM, Cisgenesis and Intragenesis, RNA-dependent DNA Methylation (RdDM), Grafting with GM Material, Reverse Breeding, Agroinfiltration, and Synthetic Genomics, but the policy also recognizes any upcoming techniques that have the potential to produce non-GM or both non-GM and GM plants as final products. Basically, the resolution involves a product-based approach in determining the presence of novel combination of genetic materials to decide whether a product is to be exempted from the existing GM regulation or not.

**Fig. 1:**
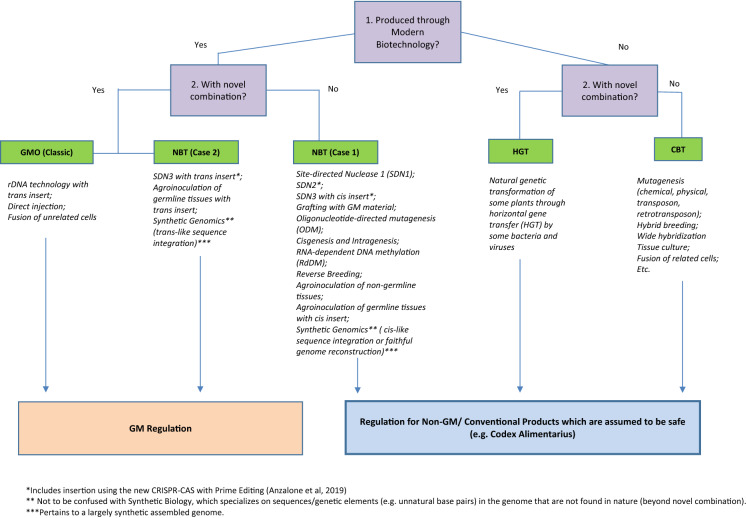
Decision tree for NBT products from the Ad Hoc TWG of the National Committee on Biosafety of Philippines (NCBP), with minor modifications. *Includes insertion using the new CRISPR-CAS with Prime Editing (Anzalone et al. 2019), ** Not to be confused with Synthetic Biology, which specializes on sequences/genetic elements (e.g. unnatural base pairs) in the genome that are not found in nature (beyond novel combination), ***Pertains to a largely synthetic assembled genome

The resolution summarizes that products of NBT/PBI can be (a) GMO, if, as defined under E.O. 514 (2006), they contain a novel combination of genetic materials obtained through the use of modern biotechnology, which “novel combination” the Ad Hoc TWG defined as a “resultant genetic combination in a living organism that is not possible through conventional breeding”; or (b) non-GMOs or conventional products, if they do not contain a novel combination of genetic materials. Only GMOs shall be regulated under the JDC No.1, whereas their non-GM counterparts are not regulated under the JDC1 but are still subject to regulations normally being applied to conventional plant products. To facilitate understanding of what techniques are covered by the policy, a decision tree was provided by the Ad Hoc TWG (Fig. 1). It is also important to note that ‘Synthetic Genomics’ and not Synthetic Biology was included in the list of NBTs. The former refers to a largely synthetic assembled genome, which may use a natural DNA sequence as template/reference, whereas the latter involves sequences/genetic elements in the genome that are not found in nature.

It is expected that once the NCBP has approved the resolution, the DA shall immediately issue specific guidelines (e.g. certificate of non-coverage under JDC No.1) and take the lead in evaluating and monitoring plant and plant products derived from NBT/PBI. As for the Ad Hoc TWG, one recommendation is for DA to adopt the “20-bp rule” (adapted from report of the *Joint Research Centre, European Commission, 2011)*, which denotes that any exogenous DNA sequence insertion that may arise from the use of NBTs must be less than 20 bp to be exempted from GM regulation. Exemptions can also be extended to longer insertions provided that the sequence is homologous or from a cross-compatible species (e.g., cisgenic). The recommendation is aimed at clearly defining, from a statistical and biological perspective, the shortest possible insertion of exogenous DNA that can potentially lead to the formation of novel combination of genetic material, which can trigger the existing GM regulation.

## International considerations

### The convention on biological diversity and the Cartagena protocol on biosafety

The regulations of most countries concerning research and development of genetically engineered organisms are based on the provisions of the Cartagena Protocol on Biosafety (CPB; CBD Secretariat, 2000). The CPB’s website also provides a resource called the Biosafety Clearing House, that is intended to compile regulatory decisions, biosafety information, and other related information contributed by Parties, other governments, and relevant organizations (http://bch.cbd.int). As of this writing, no information relating to GEd has been submitted by any Party, while three relevant organizations have submitted background information.^4^Under the Cartagena Protocol on Biosafety, the focus of discussions has been the adequacy of existing risk assessment paradigms as embodied in Annex III of the CPB, to deal with GEd. Thus, at the ninth COP/MOP, held in 2018 in Sharm El-Sheikh, Egypt, the CPB called for:

…broad international cooperation, knowledge sharing and capacity- building to support, inter alia, Parties in assessing the potential adverse effects on the conservation and sustainable use of biodiversity from living modified fish and other living modified organisms produced through new developments in modern biotechnology, including living modified organisms developed through genome editing and living modified organisms containing engineered gene drives… (Secretariat of the Convention on Biological Diversity 2020).

Under the CPB, no decisions regarding GEd has yet been agreed upon that would guide Parties to shape their domestic legislation.

While genetically engineered organisms have primarily been dealt with under the Cartagena Protocol on Biosafety, GEd has also been discussed under the parent treaty, the Convention on Biological Diversity (CBD; United Nations 1992). GEd techniques are listed as tools of synthetic biology by the CBD (Scott et al. 2015), although these techniques are not mentioned as representing a gap in oversight with respect to the provisions of the convention (Schiele et al. 2015). GEd is mentioned as one of the recent technological developments of note in online forum discussions and deliberations of the Ad Hoc Technical Expert Group (AHTEG) on Synthetic Biology (Ad Hoc Technical Expert Group on Synthetic Biology 2017). This area of research was also listed as in need of attention in order for the Convention to remain aware of technological developments in the field (Secretariat of the Convention on Biological Diversity 2018). Therefore, beyond the general provisions relating to synthetic biology in general, GEd has not emerged as a focus area for the CBD.

In the absence of a global unifying approach to the regulation of GEd, countries and regions develop their own policies and regulatory approaches, as best suits their national and regional goals and priorities. As is evident in the description of the developments in various geographies that are presented elsewhere in this paper, this current flexibility represents a challenge for researchers and product developers, yet also allows signatories to the CBD and the CPB to craft regulations that suit their particular balance between different socio-economic prerequisites, technological development and public safety.

### Regulatory impacts on international trade and innovation

The resulting different regulatory processes in different countries can have large impacts on trade and innovation. They can shape which products are developed, which products are available for farmers to grow, and what types of products are available for consumers. In the early days of the development of GE crops, there was much made of the different types of products that could be developed and made available to farmers and consumers; many of these promises for GE crops went unfulfilled. In general, there was a lack of GE products with consumer-focused traits.

The role that domestic and international regulations have played in inhibiting agricultural innovation and the development of consumer-focused traits has been greatly under-appreciated. If the costs of regulatory processes are high and the timelines and outcomes are unpredictable, the development process is both risky and expensive (Ludlow et al. 2014; Smyth and Lassoued 2019; Zimny et al. 2019; Zimny and Eriksson 2020.). These costs and uncertainties have limited the types of GE traits that have come to market, as well as the types of GE crops. With few exceptions, only those crops and traits that can provide a high return on investment are submitted into the regulatory system. The two case-studies below illustrate these phenomena. We chose the USA and Argentina for illustration as both have well-developed regulatory frameworks in place and have significant experience with cultivating GE crops.

*United States Case Study*: In the United States, for example, the petition process put in place by USDA 7CFR part 340 (1987, 1993) had 166 applications as of August 2020, with 122 of these applications from major plant biotechnology companies, while only 6 submissions were from government or academic institutions (USDA-APHIS Petitions for Determination of Nonregulated Status Website).

Of the 129 deregulated crops, 72% were for herbicide tolerant or insect resistant traits. When USDA put in place their “Am I Regulated?” process, the situation changed dramatically. As of September 3, 2020, USDA has responded to 157 “Am I Regulated?” letters of inquiry (NB: this program was phased out with the SECURE rule) and fewer than 5% of the inquiries were from major biotechnology companies while over one third have been from government or academic institutions (USDA “Am I Regulated?” website). The diversity of types of traits and the types of organisms modified has increased as well. This experience demonstrates the enormous impact that regulatory processes can have on product development.

*Argentina Case Study*: In Argentina, the decision of whether a product of a NBT is classified as a genetically engineered organism and therefore subject to more extensive requirements for approval of these products, has clear consequences for the type of entity that successfully brings a product to market. In that country, 90% of products that are classified as genetically engineered are introduced by foreign multinational companies, while 91% of products classified as non-genetically engineered are brought to market by local companies and public research and foreign small and medium enterprises—59% and 32%, respectively (Whelan et al. 2020).

The regulatory processes in other countries can also have a large impact on what products are grown by farmers in their own country, as agricultural exports can be an important part of their market. If products are not allowed in countries that are key trading partners, then this limits the ability and willingness of farmers to grow those crops domestically. International agreements can help increase harmonization across regulatory processes in different countries. For example, the United States-Mexico-Canada Agreement includes a chapter on biotechnology, which addresses concerns associated with trade of products of agricultural biotechnology and the three countries have agreed to establish a Working Group for Cooperation on Agricultural Biotechnology, with a goal to enhance information exchange and cooperation on trade and regulatory policy matters associated with agricultural biotechnology.

Argentina, United States, Canada and several other countries are well aware of trade impact due to asynchrony, asymmetric and non-scientific regulatory approaches for agricultural biotechnology, including experiences with genetically engineered crops and animal clones (Whelan and Lema 2019). To mitigate these problems, these countries have engaged in intense exchanges with like-minded and importing countries, in order to facilitate regulatory alignment and compatibility, as well as promote science-based regulation for these products that would allow their safe and effective use (and trade of derived products) at a global level.

In 2018, Argentina led efforts for release at the WTO of an International Statement on Agricultural Applications of Precision Biotechnology (USDA 2018b), which has received support from 14 countries^5^ and the Secretariat of the Economic Community of West African States. This Statement encourages “cooperative work by governments to minimize unnecessary barriers to trade related to the regulatory oversight of products of precision biotechnology, including the exploring of opportunities for regulatory and policy alignment” and promotes “constructive dialogues among trading partners and agricultural stakeholders on potential trade issues related to precision biotechnology, so as to support open and fair trade and encourage research and innovation.” (WTO 2018).

## Conclusion

The various potential products of GEd carry the promise to contribute to solving many of the great challenges of the twenty-first century, from medical and health issues to food and agricultural production. This may certainly be one of the reasons why the 2020 Nobel prize in Chemistry was awarded to Emmanuelle Charpentier and Jennifer Doudna for their discovery and development of one of the most popular GEd tools; CRISPR-Cas (https://www.nobelprize.org/prizes/chemistry/2020/summary/).

Regulatory policy cannot keep pace with the fast-moving scientific advances. To name just some of the challenges: the speed at which new technologies are being developed, new technologies not fitting into old regulatory definitions and paradigms, difficulties with international coordination, lack of harmonized definitions and laws, lack of public understanding and trust, lack of regulatory certainty for developers, lack of political will, and regulatory policies taking longer to put in place than the uptake of breakthroughs in the global scientific community. Regulatory and policy officials are frequently tasked with the sometimes conflicting goals of ensuring public and environmental safety while addressing public perception and expectations and doing so without slowing down innovation.

A number of scientific societies, regulatory agencies and other relevant organizations around the world have investigated various regulatory, safety and policy issues surrounding GEd techniques, issuing science-based opinions and proportionate recommendations to policymakers formulating regulations (see, e.g., ASSAf 2017; CAST, 2018; EASAC, 2015; EFSA 2012, 2015, 2020; FSANZ 2019b; JRC 2011; Leopoldina 2015; USDA 2018a, VIB 2018). Some of these studies, scientific opinions, and statements (and their recommendations) are discussed in the relevant country sections, above. The common conclusions in these opinions include imposing regulatory scrutiny based on the documented risks of the product, rather than on the process used to breed them, and that many products of GEd may not warrant additional regulation beyond those required for conventional plants, especially if they could have been generated using ‘conventional’ methods of breeding.

Many countries are still in the process of developing regulatory approaches for products of GEd plants, so the opportunity remains for enhancing global regulatory coordination. The positive consequences for sustainable agricultural innovation and international trade could be considerable. Among many countries that have already finalized their GEd regulatory approaches some positive alignment is emerging in terms of using a “case-by-case” approach (offering the ability to balance science-based risk management and societal requirements) using a “novel combination of genetic material” as the GMO regulatory threshold (offering the ability to distinguish between GMOs and non-GMOs).

We hope this paper has provided insight into the diverse incipient regulatory policies governing GEd agricultural products in a range of countries and jurisdictions. We hope that these insights spurs action leading to increased collaboration and coordination among countries to better align regulatory processes and enhance coordination of approaches globally.

## Supplementary Information

Below is the link to the electronic supplementary material.

Supplementary file1 (DOCX 32 kb)

## Footnotes

1 Panel Reports, *European Communities—Measures Affecting the Approval and Marketing of Biotech Products*, WT/DS291/R; WT/DS292/R; WT/DS293/R (September 29, 2006, adopted November 21, 2006), available at https://www.wto.org/english/tratop_e/dispu_e/cases_e/ds291_e.htm (accessed 5 December 2020).

2 Note that relative to USDA requirements under SECURE, once a plant with a PIP is registered by the EPA, USDA exercises regulatory discretion to cede regulation to EPA, so long as the registration remains current. Under the previous USDA regulations, USDA had regulatory authority on PIPS as long as field releases were < 10 acres.

3 “Regulatory trigger” refers to the defining element that prompts regulation under a specific regulatory framework or act. Although this concept is prominent in the product vs process debate in the GMO-context, it is rarely used elsewhere in legal interpretation. The need for defining the exact “trigger” for regulation evidently only arises when the jurisdiction of a specific act, as evident from an encompassing, contextual interpretation of its aims, definitions, provisions, regulations, etc., is disputed.

4 As of September 28, 2020, organizations submitting information are Imperial College London, Biofuelwatch, and African Centre for Biosafety. The BCH database http://bch.cbd.int/database/submissions/ was searched for the term “editing”.

5 Argentina, Australia, Brazil, Canada, Colombia, the Dominican Republic, Guatemala, Honduras, Jordan, Paraguay, the Philippines (joined in 2020), the United States, Uruguay and Viet Nam.
