# Supplementary material for: Regulatory approaches for genome edited agricultural plants in select countries and jurisdictions around the world
Source: Transgenic Res. 2021 May 10;30(4):551–84. doi: 10.1007/s11248-021-00257-8 (PMC8316157; doi:10.1007/s11248-021-00257-8)
Supplement: Supplementary file 1 — Supplementary file1 (DOCX 32 kb) [file 11248_2021_257_MOESM1_ESM.docx]

**SUPPLEMENTAL INFORMATION:**

**United States:**

**Coordinated Framework for Regulation of Biotechnology**

In 1986, the U.S. Office of Science and Technology Policy (OSTP), an office of the White House, published the Coordinated Framework for Regulation of Biotechnology (OSTP 1986), a regulatory policy for agricultural research and products of biotechnology that drew on existing laws to establish a forward-looking regulatory framework.

The Coordinated Framework established policies that have been fundamental to the U.S. regulation of products of biotechnology: 1) existing U.S. regulatory statutes are sufficient to accommodate the regulation of products of biotechnology and therefore there was no need for the United States to enact a biotechnology-specific regulatory framework of new statutes or create new regulatory agencies; 2) regulation should be science-based and conducted on a case-by-case basis by applying precedent and advances in knowledge; 3) the regulation of products of biotechnology would be coordinated among existing U.S. regulatory agencies using already enacted statutes that would be applied to products of biotechnology. The Coordinated Framework laid out the roles and responsibilities of the U.S. regulatory agencies and identified lead agencies for different types of biotechnology products.

As a result of these policies, the Coordinated Framework promoted coordination and cooperation among relevant agencies. For agricultural products of biotechnology, the lead agencies were identified as the United States Department of Agriculture (USDA), the Environmental Protection Agency (EPA), and the Food and Drug Administration (FDA). Thus, this discussion focuses on these three agencies, their statutory authority and their administrative regulations and procedures related to plants that are the product of agricultural biotechnology.

The Coordinated Framework states that Federal regulatory oversight would be risk-based and focus on the characteristics of the biotechnology product, rather than on the genetic modification technique used to create it. OSTP issued updates of the Coordinated Framework in 1992 and most recently in 2017 (OSTP 1992, 2017). Recent updates to the Coordinated Framework and U.S. regulatory processes to accommodate crops produced via new biotechnologies such as genome editing, were prompted by two separate presidential actions. [See Article for more details]

**United States Department of Agriculture (USDA)**

USDA’s Animal and Plant Health Inspection Service (APHIS) is responsible for protecting U.S. agriculture, environment and economy from pests and diseases. APHIS has regulatory authority over products of biotechnology that are plants through the Plant Protection Act (PPA), which obligates USDA to protect plant health. Plant pest risk is the potential for direct or indirect injury to, damage to, or disease in plants or plant products resulting from introducing or disseminating a plant pest, or the potential for exacerbating the impact of a plant pest.

In 1987, APHIS issued regulations under PPA authority over the importation, inter-state movement, or release into the environment of any “regulated article” defined as 1) a genetically-engineered organism developed using r-DNA technology – primarily plants; and 2) that is or poses a risk of being a plant pest; the regulations were later updated in 1993 (APHIS 1987, 1993). These regulations encompassed any plant that contained rDNA introduced by a plant pest; or any plant containing rDNA obtained from a plant pest regardless of how the rDNA was introduced. At that time, breeders of genetically engineered plants most commonly used *Agrobacterium tumefaciens*-mediated gene transfer and vectors containing rDNA sequences of bacterial or viral origin (both plant pests) – therefore these genetically engineered plants were “regulated articles.”

In accord with these regulations, and depending on the gene-crop combination, plant developers were required to either notify APHIS or apply for a permit from APHIS for field trials or movement of genetically engineered plants. In both cases, applicants needed to comply with various measures to reasonably limit escape of the transgenes. Finally, plant developers could petition for a determination of non-regulated status of the genetically engineered plant based upon the information and data gathered in the field trials authorized by notification or permit. When APHIS granted the petition for non-regulated status on the basis that the genetically engineered plant was not and did not pose a greater plant pest risk than the conventional counterpart, then the genetically engineered plant was deregulated.

**Environmental Protection Agency (EPA)**

The EPA exercises regulatory control over biotechnology through three statutes: the Federal Insecticide, Fungicide and Rodenticide Act (FIFRA), section 408 of the Federal Food, Drug and Cosmetic Act (FFDCA), and the Toxic Substances Control Act (TSCA). The assessment of genetically engineered crops under FIFRA focuses on the pesticidal property rather than the crop itself.

**FIFRA**: The statutory language under FIFRA in part defines a pesticide as any substance intended to prevent, destroy, repel or mitigate any pest. FIFRA gives EPA the authority to regulate pesticides, including substances that plants produce for protection against pests, known as plant-incorporated protectants (PIPs). In 2001, EPA published regulations that defined a PIP as “a pesticidal substance that is intended to be produced and used in a living plant, or in the produce thereof and the genetic material necessary for production of such a substance” and subject to regulatory oversight under FIFRA (EPA 2001). A pesticide can only be registered if it can be shown that it will cause “no unreasonable adverse effects to human health or the environment.” Some PIPs are exempted from regulation under FIFRA. EPA has determined that the naturally occurring PIPs present in plants or moved between plants through conventional breeding do not need to be regulated; they are specifically exempted from FIFRA registration requirements based on a history of safe use in agriculture and in food and feed products (US EPA, 2001). In general, when EPA grants a pesticide registration, the registration is in effect for fifteen years at which time the registrant must go through a registration renewal procedure; many PIPs are reassessed after a few years rather than the maximum 15 years. The specific technology used to modify the DNA of a plant is not the relevant criterion in determining whether the substances comprise a PIP. Rather, EPA advises that the intended use and claims made for preventing, destroying, repelling or mitigating a pest determine whether that particular use is pesticidal. Thus, EPA regulation of PIPs is not based on the specific technology used to modify the plant.

**FFDCA**: EPA regulates the safety of any residual amounts of a pesticide or substances resulting from the use of the pesticide, including PIPs, on the crop or food, that occur in or on food or feed under section 408 of FFDCA. EPA may establish a tolerance (maximum residue levels) or tolerance exemption that applies to both domestic and imported foods only if there is a reasonable certainty that no harm will result from aggregate exposure to residues of the pesticide in food for humans or animals. Under a tolerance exemption, there are no numerical limitations on the amount of pesticide chemical residues that may be in or on food. EPA will not issue a FIFRA registration for a PIP in a plant used for food or feed unless an FFDCA tolerance exemption for residues of that PIP is in place.

NEPA does not apply to the EPA FIFRA review because FIFRA reviews are considered to be equivalent to those required under NEPA. In other words, EPA FIFRA review considers if there is a significant impact on the human environment of any pesticide.

**Food and Drug Administration (FDA)**

The FDA has regulatory control over food from plants developed via biotechnology and all forms of genetic modification, including conventional breeding, under the Federal Food, Drug & Cosmetic Act (FDCA).

**“Voluntary” consultation**: Sellers have the obligation to ensure that the food and feed they sell is safe and legal for human and animal consumption, regardless of the method or technology used to produce the food or feed. While FDA has no premarket authority over whole foods (as in contrast with food and color additives), it has power to take enforcement action against food that is not safe or legal. Hence, if a seller has any doubt over the safety of their product, they should consult with FDA. In addition, buyers, shippers, or traders of food and feed products may require FDA consultation before agreeing to buy or ship a product.

FDA’s role to ensure foods and feeds are safe and legal exists regardless of the method or technology used to produce the food or feed. In 1992, FDA recommended that a consultation process be used for foods derived from biotechnology (FDA 1992), whereby producers of genetically engineered crops and ingredients voluntarily consult with FDA about their genetically engineered crops prior to commercial release. In the voluntary consultation process, producers provided information to FDA to show that their genetically engineered crops are as safe as conventional equivalents. So far, biotechnology developers have participated in this consultation on every genetically engineered crop introduced into the US market, and the first edited crop on the market (a soybean with altered oil composition) also went through this process. FDA does not formally ‘approve’ the product, but instead issues a “the consultation is complete” letter at the end of the process.

**Disclosure or labeling of foods containing bioengineered content**

In July 2016, the United States Congress passed the National Bioengineered Food Disclosure Standard, which required the establishment of a national mandatory standard for disclosure for foods that are or maybe bioengineered (NBFDS 2016). Congress tasked the USDA-Agricultural Marketing Service (AMS) with drafting implementing standards. USDA-AMS released those final standards – the National Bioengineered Food Disclosure Standard (NBFDS) – in December 2018 (USDA-AMS 2018). USDA-AMS is a marketing division of USDA and they have stressed that disclosure standard is for purposes of consumer information and do not say or imply, explicitly or implicitly, anything about the nutrition, safety, or environmental attributes of the disclosed food.

AMS incorporated the statutory definition of bioengineering into its regulatory definition, reading as follows for human food (animal feed is expressly excluded by the statute): “(A) that contains genetic material that has been modified through *in vitro* recombinant deoxyribonucleic acid (DNA) techniques; and (B) for which the modification could not otherwise be obtained through conventional breeding or found in nature.” Food manufacturers who intentionally use a detectable bioengineered ingredient, or who have a food with an inadvertent or technically unavoidable bioengineered ingredient detectable at a level of 5% or greater per ingredient, must disclose that the human food contains “bioengineered” content. Highly-refined foods (e.g., oils or sugar) are not considered bioengineered foods under this standard so long as: (1) those foods do not contain detectable modified genetic material and (2) the entity maintains records to demonstrate modified genetic material is not detectable. Very small food manufacturers (those with less than $2.5 million in total sales) are exempt from the disclosure requirements. The USDA-AMS regulations require disclosure beginning January 1, 2022.

**Canada:**

**Canada’s Regulatory Framework**

Canada utilizes a metric of substantial equivalence in its risk assessment process, that is, if the risks of genetically engineered crop are substantially equivalent to those of non-GE, conventional crop production, then the genetically engineered crop variety is approved for commercial release. All crop and food production, regardless of the process used, has an impact on the environment and ecology and substantial equivalence serves as a means of ensuring that the impacts from the production of genetically engineered crops do not differ from other crop production. Canada does not regulate based on the process use to create new plant varieties, such as is done in the European Union, but regulates instead the product of plant breeding. The regulatory framework that was developed in Canada through consultations between academic scientists, private sector plant breeders and scientists and federal regulators, assesses the risk of plants with novel traits (PNTs). Any plant breeding process, or even foreign introductions, may create a product that is deemed to be novel, such as with the development of insect resistant corn through genetic engineering or herbicide tolerant wheat through mutagenesis. Canada has approved the third highest number of traits of the 70 countries that have approved GM varieties, with 429 approvals. Of this, 147 approvals have been for food, 138 for feed and 144 for cultivation. Cultivation approvals are slightly lower, due three genetically engineered events being approved for food import, that would not be grown in Canada, such as Golden Rice or virus resistant papaya. Additionally, genetically engineered salmon have been approved and would not be commercially cultivated in the sense that a genetically engineered crop would be.

The regulation of PNTs in Canada is governed by three federal acts, Seeds Act, Feeds Act and Food and Drugs. The Seeds Act and the Feeds Act fall under the mandate of the Canadian Food Inspection Agency (CFIA) while the Food and Drugs Act is under the mandate of Health Canada. All three of these acts existed in Canada prior to the development of genetically engineered crops and were revised to undertake science-based risk assessments of genetically engineered varieties. This process required six years of consultation, discussion and knowledge sharing, beginning in 1988 and concluding in 1994. The workshops and conferences that were held as part of the PNT development process, relied heavily on the globally accepted risk assessment methodology that was developed by the Organisation for Economic Cooperation and Development (OECD), in consultation with scientists from around the globe (OECD 1986). A cumulative total of 1,200 references were used in Canada, the USA and Europe in the development of regulatory frameworks for genetically engineered crops. The PNT framework is based on 56 scientific publications, documents and commissioned reports (Gleim and Smyth 2018).

The Seeds Act governs the risk assessment factors of gene flow, invasiveness, weediness, impacts on non-target organisms and other potential negative impacts on biodiversity. Gene flow assesses the probability of a genetically engineered plant pollinating a weedy relative and what the viability of any resulting progeny might be. For example, with genetically engineered canola, it was determined there were three potential weedy relatives, but research determined none of these weed species were capable of surviving the cold temperatures of prairie winters (Smyth et al. 2002). The invasiveness of a plant examines the plant’s ability to adapt and spread to regions where they have not previously grown and that lack natural predators. Weediness involves an assessment of the plant to become a weed in other crops. With regard to genetically engineered canola, weediness was found to be no different than non-GE canola as canola was included on the list of top 10 weeds for many years prior to the commercialization of genetically engineered canola. The assessment of risks on non-target organisms, for example involves assessing the risks of a genetically engineered crop impacting organisms such as insects (via plant consumption), wildlife (through unintended plant or seed consumption) and impacts on aquatic life (through unintended seed dispersal, such as by wind). Finally, a catch-all assessment is done for any potential for risks to any biodiversity aspects that would not be captured by the risk assessments on gene flow, invasiveness, weediness, impacts on non-target organisms.

The Feeds Act governs the risk assessment factors in animals regarding allergenicity, toxicity, digestibility and dietary exposure. Risk assessment of allergenicity examines how rapidly the novel proteins that have been added to the plant via genetic engineering break down in the gut of animals, which quantifies whether the protein could pose the potential to be an allergen. Similarly, toxicity risk assessments examine data on whether the PNT could create a toxic effect in any livestock that intentionally, or unintentionally, consumes the PNT variety. It also assesses whether the protein is expressed equally throughout all plant matter and seed. Risk assessment involving digestibility examines whether there are any effects on an animal’s ability to digest either the plant or resulting seed, following consumption. Essentially, it examines whether any organic digestion function is impacted. The risk assessment pertaining to dietary exposure quantifies whether there is a level of consumption that would create a health concern for any livestock.

The Food and Drugs Act governs risk assessment factors in humans involving allergenicity, toxicity, metabolization, nutrition and dietary exposure. The risks the Feeds Act assesses in livestock are largely replicated by the assessments undertaken by the Food and Drugs Act for human consumption. Assessments for allergenicity and toxicity involve the same assessments for human consumption as is the case for livestock, that is how rapidly novel proteins break down upon consumption and whether this changes any legitimate risk regarding the potential for triggering an allergic reaction or posing as a toxin. Metabolization examines any potential for risks to develop in humans once the genetically engineered product has been consumed. Nutrition risk assessment assesses whether there are any changes in the nutritional composition of the final product, that might impact human health. Similar to livestock, dietary exposure examines whether there are consumption levels that could adversely impacts humans.

**Australia and New Zealand:**

**Supplemental Information:**

**Table 1.** Details of the regulatory agencies with responsibilities for the regulation of GM products in Australia

| Agency | Products regulated | Relevant Legislation/Agreements |
| --- | --- | --- |
| Office of the Gene Technology Regulator (OGTR) | All ‘dealings’ with GMOs | *Gene Technology Act 2000*  *Gene Technology Regulations 2001*  *Gene Technology Agreement 2001* |
| Food Standards Australia New Zealand (FSANZ) | Food for human consumption | *Food Standards Australia New Zealand Act 1991*  *Food Standards Australia New Zealand Regulations 1994*  *Imported Food Control Act 1992*  *Food Standards Code* |
| Therapeutic Goods Administration (TGA) | Human therapeutic goods | *Therapeutic Goods Act 1989*  *Therapeutic Goods Regulations 1990* |
| Australian Pesticides and Veterinary Medicines Authority (APVMA) | Agricultural chemicals and veterinary medicines | *Agricultural and Veterinary Chemicals (Administration) Act 1992*  *Agricultural and Veterinary Chemicals Code Act 1994.* |
| Australian Industrial Chemicals Introduction Scheme (AICIS) | Industrial chemicals | *The Industrial Chemicals Act 2019* |
| Department of Agriculture, Water and the Environment (DAWE) | Import and export | *Biosecurity Act 2015* |

**Table 2.** Current status of moratoria in Australia

| State/Territory | Legislation | Current Status |
| --- | --- | --- |
| Australian Capital Territory | *Gene Technology (GM Crop Moratorium) Act 2004* | Act in force. Agriculture Minister has the ability to make Orders to designate areas GM free and/or prohibit or restrict the cultivation of a GM food crops. Orders currently in place preventing the cultivation of GM canola |
| New South Wales | *Gene Technology (GM Crop Moratorium) Act 2003 No 12* | Act is in force, but due to expire 1 July, 2021. Provides a regime to regulate  the commercial cultivation of licensed GM food plants, and the conduct of experiments on licensed GM food plants for marketing purposes. Currently no bans in place |
| Northern Territory | There is no GM crop moratorium in the Northern Territory, however, there is currently no commercial cultivation of GM crops | |
| Queensland | Queensland has never had a moratorium on GM crops | |
| South Australia | *Genetically Modified Crops Management Act 2004* | Act is in force. Provides a regime to regulate:  the commercial cultivation of licensed GM food plants and designate areas GM free. Currently under review. |
| Tasmania | Tasmania remains with a moratorium on the commercial release of GMOs indefinitely | |
| Victoria | *Control of Genetically Modified Crops Act 2004* | Act in force. Agriculture Minister has the ability to make Orders to designate areas of the State GM free and/or prohibit or restrict the cultivation of GM crops. Currently no bans in place |
| Western Australia | *Genetically Modified Crops Free Areas Act 2003; Repealed October 2016* | Legislation no longer in place. |

| **Box 1** Main laws governing the use of genetically modified organisms in New Zealand |
| --- |
| - Medicines Act 1981 - Official Information Act 1982 - Biosecurity Act 1993 (including Ministry for Primary Industries (MPI)/Environmental Protection Agency (EPA) Facility Standards; MPI Import Health Standards) - Hazardous Substances and New Organisms (HSNO) Act 1996 - Customs and Excise Act 1996 - Agricultural Compounds and Veterinary Medicines (ACVM) Act 1997 - Hazardous Substances and New Organisms (Methodology) Order 1998 - Hazardous Substances and New Organisms (Low-risk Genetic Modification) Regulations 2003 - Import and Exports (Living Modified Organisms) Prohibition Order 2005, pursuant to section 3A(1) of the Imports and Exports (Restrictions) Act 1988 - Food Act 2014 |

**Japan:**

**Biosafety regulations in Japan**

In Japan, a developer of GMOs is required to receive three different approvals on food, feed, and environmental safety (that is, the impact on biodiversity) prior to commercial distribution of the products in Japan under the Food Sanitation Act (Ministry of Justice 2020a), the Feed Safety Act (FAMIC 2020), and the Cartagena Act, respectively (Ministry of Justice 2020b). In Table 1, I summarize the current relationship between the Japanese Act on the handling of living modified organisms and the related ministries and agencies. Regulation of genetic modification in Japan is governed by three ministries—namely, the Ministry of the Environment (MOE), the Ministry of Agriculture, Forestry and Fisheries (MAFF), and the Ministry of Health, Labor and Welfare (MHLW). In this section, I introduce the procedures for food safety, feed safety, and environmental impact.

| Table 1 GMOS-related Acts and competent authorities | | | |
| --- | --- | --- | --- |
|  | Food safety | Feed safety | Environmental safety |
| Act | Food Sanitation Act | Feed Safety Act | Cartagena Act |
| Risk Management | Ministry of Health, Labor and Welfare | Ministry of Agriculture, Forestry and Fisheries | Ministry of Agriculture, Forestry and Fisheries, Ministry of Environment |
| Risk Assessment | Food Safety Commission | Agricultural Materials Council, Food Safety Commission | Biodiversity Risk Assessment Committees |

・Food Safety

Under the Food Sanitation Act, GM foods are required to receive food safety approval from the MHLW prior to distribution in the Japanese market. An applicant submits a dossier for each parental GM event to the MHLW, rather than for each gene to be introduced or for each combination of the transgene and the host species. After the MHLW has conducted a thorough check of the dossier for completeness, the MHLW requests the Food Safety Commission (FSC), which falls under the Cabinet Office, to conduct an assessment for food safety. The FSC comprises primarily seven commissioners who are appointed based on their profound knowledge of food safety. As its subordinate structure, the FSC has 12 Expert Committees that operate to implement risk assessments on individual hazards such as food additives, pesticides, genetically modified foods, and so on. The actual food safety assessment for GM foods is conducted by the Expert Committee comprising university professors and scientists of national research organizations. After the committee completes the food safety assessment, the FSC opens a public comment period lasting 30 days. Having reviewed the public comments, in the absence of any new safety concern, the FSC reports the result of the food safety assessment to the MHLW, which then notifies the completion of the safety assessment in the Official Gazette.

The food safety assessment is conducted based on the Standards for the Safety Assessment of Genetically Modified Foods (Seed Plants) (FSC 2004a). The standards are built on the concept of “substantial equivalence” in accordance with the Codex Guideline for the Conduct of Food Safety Assessment of Foods Produced using Recombinant-DNA Plants (FAO 2003). Safety assessment takes into account both intended and unintended changes. More specifically, the dossier requires the inclusion of the following information:

1. properties of the host used as a counterpart in safety assessment and its differences from the GM crop
2. purposes and usages of the GM crop
3. history of safe consumption of the host plant, including information on production of harmful physiological substances
4. safety of the inserted DNA or gene(s) and gene product(s)
5. food safety of the GM crop
6. additional information when safety cannot be confirmed based on the information listed in A) to E).

- Feed Safety

GM feeds are required to receive feed safety approval from the MAFF prior to distribution in the Japanese market. The feed safety assessment is conducted based on the following two considerations, which are fundamental in ensuring feed safety:

1. safety of the GM crops for animal health;
2. safety of the animal products—such as meat, milk, and eggs produced by animals fed on the GM crop—for human health.

An applicant submits a dossier for each parental GM event to the MAFF. After conducting a thorough check of the dossier for completeness, the MAFF requests the Feed Subcommittee of Agricultural Materials Council, comprising experts, to conduct a safety assessment of the GM crops for animal health, and also requests the FSC to conduct a safety assessment of the animal products fed on the GM crop. After both the Agricultural Materials Council and the FSC have completed the safety assessment, the MAFF invites public comments over 30 days. Having reviewed the public comments, in the absence of any new safety concern, the MAFF notifies the completion of the safety assessment in the Official Gazette.

As is the case with the food safety assessment, the feed safety assessment is based on the concept of “substantial equivalence” and takes into account both intended and unintended changes. The required data and information for the feed safety assessment are similar to those required for the food safety assessment (FSC 2004b).

Since most of the GM crops are used for both food and feed, and are therefore assessed for food safety, the following three points are identified as the new potential risks to be considered in the safety assessment of the animal products fed on GM feed (MAFF 2002):

1. the possibility that a new harmful substance(s) is produced in the GM feed and transferred into animal products such as meat, milk, and eggs;
2. the possibility that the gene product(s) is converted into a harmful substance in the animal products and that harmful substance is accumulated in the animal products;
3. the possibility that the gene product(s) interacts with an animal metabolic pathway(s) and produces a new harmful substance(s).

An applicant assesses those three possibilities and submits a dossier demonstrating why such possibilities are negligible, supported by scientific rationale.

- Environmental Safety

Prior to environmental release, GM crops are required to receive Type I-use approval from the MOE and the MAFF under the Cartagena Act. Type I-use approval is for non-confined use of GM crops; that is, domestic cultivation and/or import for food, feed, and processing. Type II-use approval is for contained use in greenhouses or laboratories with containment measures. For Type I-use approval, an applicant submits a dossier for each event to the MAFF, which is the contact agency for submissions. After the MOE/MAFF have completed a thorough check of the dossier for completeness, the Ministries request the Biodiversity Impact Assessment Study Committee, comprising experts from the universities and the national research organizations, to conduct assessment of the biodiversity impact of the GM crop. The committee should consider whether the applicant has clearly considered and assessed the risk of the event with respect to the assessment endpoints of cross ability, productivity of harmful substances, and competitiveness.

After the Committee has completed the risk assessment, the Ministries jointly open a public comment period lasting 30 days. Having reviewed the public comments, in the absence of any new safety concern, the Ministries notify the completion of the safety assessment in the Official Gazette. For food and feed crops, however, the environmental safety approval is put on hold until the food and feed safety approvals have been granted. The standard operating procedures regarding the biodiversity impact assessment and management of GM crops is posted on the MAFF website (MOE 2016). USDA (2019a) provided the information that the latest status of consumption, regulation, public perception, research, development, production, and use of agricultural biotechnology in Japan. In the report, the concept of the committee’s judgement was also reported.

A unique feature of the Japanese biodiversity impact assessment process is that—even if the purpose of a Type I-use approval is for an import of a GM crop for food, feed and processing, the attachment of agronomic phenotype data in a dossier is required, with details of confined field testing conducted in at least at one location and one season in Japan. The principle of the assessment is “familiarity” of the GM crop to its conventional counterpart—that is, if the environmental impact of the GM crop on the receiving environment does not exceed that of its conventional counterpart, it is considered to be as safe as the conventional crop. This principle is the essence of the Japanese Cartagena Act.

**Brazil:**

**Brazilian Regulatory Framework overview**

Brazilian Law No. 11.105 of 24 March 2005 approved by the National Congress is known as the (Brazilian Biosafety Law, 2005) put an end to the legislative controversy surrounding GMOs in the country. This law was a comprehensive and a complementing revision of a previous biosafety law of 1995 and determined the general rules for research and commercial activities with GMOs in Brazil. The principles used to elaborate this law were to encourage scientific advances in the areas of biosafety and biotechnology, protection of life, human health, animal and plant health and compliance with the precautionary principle for protection of the environment, according to Cartagena Protocol ( Secretariat of the Convention on Biological Diversity, 2000). It established the National Biosafety Council (CNBS), restructured the CTNBio and proposed the Brazilian Biosafety Policy. Its purpose and scope were to provide safety standards and inspection mechanisms for the construction, cultivation, production, handling, transportation, transfer, import, export, storage, research, environmental release and commercialization of GMOs and their by-products. The legislation requests that all public and private organizations, national or foreign, that conduct activities or research projects in Brazil, under the description of Law no 11.105, require a Biosafety Quality Certificate issued by CTNBio before starting any activity. CTNBio through its Normative Resolutions is responsible for establishing the biosafety guidelines for subjects of its competencies. Among its prerogatives and in consequence of the development of science and technology in the world, the law mandates the CTNBio to evaluate how new technologies can impact the environment, and human and animal health in the country and then, if necessary, authorize the commission to propose regulations for these new technologies. CTNBio consists of 27 Brazilian citizens, appointed by the Minister of Science and Technology (S&T), who have recognized technical competence, outstanding scientific performance and knowledge, an academic PhD, and outstanding professional activity in the fields of: Biosafety, Biotechnology, Biology, Human Health, Animal Health, and Environment. The list of names to be chosen by the Minister of S&T consists of nominations from the other ministries of the Brazilian government and also specialists named by a committee of the Brazilian Academy of Science.
